# Assessing Diversity of DNA Structure-Related Sequence Features in Prokaryotic Genomes

**DOI:** 10.1093/dnares/dst057

**Published:** 2014-01-09

**Authors:** Yongjie Huang, Jan Mrázek

**Affiliations:** 1 Institute of Bioinformatics, University of Georgia, Athens, GA 30602, USA; 2 Department of Microbiology, University of Georgia, Athens, GA 30602, USA

**Keywords:** sequence patterns, Z-DNA, DNA curvature, sequence repeats, palindromes

## Abstract

Prokaryotic genomes are diverse in terms of their nucleotide and oligonucleotide composition as well as presence of various sequence features that can affect physical properties of the DNA molecule. We present a survey of local sequence patterns which have a potential to promote non-canonical DNA conformations (i.e. different from standard B-DNA double helix) and interpret the results in terms of relationships with organisms' habitats, phylogenetic classifications, and other characteristics. Our present work differs from earlier similar surveys not only by investigating a wider range of sequence patterns in a large number of genomes but also by using a more realistic null model to assess significant deviations. Our results show that simple sequence repeats and Z-DNA-promoting patterns are generally suppressed in prokaryotic genomes, whereas palindromes and inverted repeats are over-represented. Representation of patterns that promote Z-DNA and intrinsic DNA curvature increases with increasing optimal growth temperature (OGT), and decreases with increasing oxygen requirement. Additionally, representations of close direct repeats, palindromes and inverted repeats exhibit clear negative trends with increasing OGT. The observed relationships with environmental characteristics, particularly OGT, suggest possible evolutionary scenarios of structural adaptation of DNA to particular environmental niches.

## 1. Introduction

Prokaryotic genomes are extremely diverse in terms of their nucleotide and oligonucleotide composition as well as presence of various forms of sequence repeats and patterns that can affect physical properties of the DNA molecule. For example, Ussery *et al*.^1^ analysed oligopurine/oligopyrimidine runs and alternating purine/pyrimidine patterns in prokaryotic genomes and reported large differences among different organisms. Alternating purine/pyrimidine patterns promote Z-DNA conformation, whereas oligopurine/oligopurimidine runs can facilitate formation of A-DNA or H-DNA.^2^ They found that differences among different prokaryotes were related to optimal growth temperature (OGT) and pathogenicity, possibly as a result of structural adaptations of DNA of these organisms.^3^ In another example, potential G-DNA-forming sequences were found to occur at vastly different frequencies in different prokaryotic genomes.^4^ The amount of intrinsic DNA curvature in promoter regions was found to be related to OGT.^5^ Our own analysis of more than 1000 prokaryotic chromosomes suggested a large variance among different genomes in DNA curvature-related 10–11 bp sequence periodicity, which could reflect differences in chromosomal structure.^6^ The presence of long simple sequence repeats (SSRs) in a genome is strongly correlated with the organism's dependence on a eukaryotic host.^7^ These results indicate a significant variance of general DNA properties among different prokaryotes, which in some cases appear to be related to the organisms' habitats and lifestyles.

Advances in DNA sequencing technologies over the past decades led to a situation where complete genomes are available for many microbes about which very little is known apart from the information that can be derived from the genomic DNA sequence. In particular, despite the diversity of DNA properties mentioned above, our knowledge about chromosome structure and organization in the cell is limited to studies of a few model organisms. While it is reasonable to assume that the general model of bacterial nucleoid composed of dynamic, supercoiled DNA loops stabilized by nucleoid-associated proteins is probably universal,^8,9^ the differences in DNA sequence properties suggest that subtle variations may exist among bacteria with completely sequenced genomes. Such differences in physical characteristics of the chromosomes could play roles in the organisms' physiology and adaptations to their particular environments.

We present a survey of local sequence patterns in prokaryotic genomes with a potential to generate local irregularities in DNA structure. Our goal was to assess diversity of the prokaryotic genomes in terms of abundance of sequence patterns indicative of possible structural transitions in the DNA molecule. While physiological functions of sequence patterns promoting non-canonical DNA conformations and sequence patterns promoting conformational transitions in DNA are not well understood, there is increasing evidence that they can play important roles. For example, SSRs have been implicated in phase variation, a mechanism that promotes reversible switching of phenotypes.^10,11^ Palindromes and close inverted repeats form stem-loop structures in RNA, which can function in transcription terminators and riboswitches, and they can promote formation of cruciform structures in DNA, which influence replication, regulation of gene expression, and recombination.^12^ Close direct and inverted repeats have mutagenic effects on DNA.^13–16^ Specific guanine-rich patterns can promote formation of quadruplex G-DNA.^17–19^ The G-DNA formation in telomeres is well documented, but G-DNA may also play a role in gene expression, replication, recombination, and integration of viruses.^19–23^ Alternating purine–pyrimidine patterns can under favourable conditions promote transitions to the Z-DNA conformation, while oligopurine/oligopyrimidine runs promote formation of A-DNA or triple-stranded H-DNA.^2,24–28^ Transient formation of left-handed Z-DNA can influence transcription and promote genome instability,^28–31^ and indirect evidence suggests similar roles for triple-helical H-DNA.^26,32^ Intrinsic DNA curvature is largely related to periodic spacings of A-tracts and can influence DNA–protein interactions in regulatory regions, aid DNA compaction in the nucleoid, and possibly promote a particular mode of supercoiling.^5,33,34^ Although the physiological significance of the unusual DNA conformations in different organisms is still poorly understood, it is reasonable to ask how common the sequence patterns that promote their formation are in different genomes because their unusually high or low occurrence may indicate its functional importance.

Our present work differs from earlier similar surveys not only by investigating a wider range of sequence patterns in a large number of genomes but also by using a more realistic null model to assess significant anomalies. Significant deviations from expected pattern occurrences could indicate that the pattern *per se* is subject to selective constraint and therefore functionally significant in a given organism but the pattern over- or under-representation could also arise as a tolerated artefact of other biases affecting the DNA sequence. Our null model reflects the genome-specific nearest neighbour preferences, codon biases, and heterogeneity of the DNA sequence; therefore, deviations from expected occurrences are more likely to reflect their functional significance of the investigated sequence patterns. We interpret our results in terms of relationships with organisms' habitats, phylogenetic classifications, and other characteristics.

## 2. Materials and methods

### 2.1. Data sets

Complete prokaryotic genomes were downloaded from the National Center for Biotechnology Information (NCBI) ftp server (ftp://ftp.ncbi.nih.gov/genomes/Bacteria/). Pattern representations were assessed for the complete genomes, as well as for 1 Mb segments randomly selected from each genome. The purpose of the latter approach was to reduce the effect of statistical artefacts from comparing sequences of different sizes. The results presented in this paper are based on the 1 Mb segments, while the data for the complete genomes are shown in the Supplementary data (Supplementary Tables S5–S7). Genomes smaller than 1 Mb were excluded from the analysis. Our final data set included 1424 complete genomes of 941 species, 519 genera, and 37 phyla or subphyla (Supplementary Table S1). We used the existing annotation (the ‘CDS’ keywords) to differentiate protein-coding and non-coding sequences.

The OGT and oxygen requirement classifications for each genome were obtained from the Genomes Online Database (http://www.genomesonline.org).^35^ Among the 1424 complete genomes, 1378 genomes have the OGT classification available and 1304 genomes have the oxygen requirement classification available. To eliminate sampling biases towards genera represented by many completely sequenced genomes, we performed statistical assessments at the level of genera rather than individual genomes. Accordingly, we apply a single classification to each genus, which is determined by the majority of species within the genus. This procedure yielded OGT classification for 498 genera (including 18 psychrophiles and psychrotolerant organisms, 382 mesophiles, 72 thermophiles, and 26 hyperthermophiles) and oxygen requirement classification for 465 genera (159 anaerobes, 202 aerobes, 95 facultative, and 9 microaerophilic organisms).

### 2.2. Patterns of interest

Table 1 provides a list of sequence patterns whose occurrences in prokaryotic genomes were investigated in this work. The patterns were selected based on the available information about sequences that promote various forms of structural transitions or mutations in DNA under favourable circumstances. However, because exact rules governing structural transitions in DNA are not fully understood, we use multiple forms of similar patterns, in most cases pertaining to varying length of the pattern and number of tolerated mismatches. The specific parameters used in the definition of the patterns were in part dictated by the size of the analysed genomes in order to obtain sufficient data sample for statistical evaluations.

**Table 1.** List of sequence patterns investigated in this work

Pattern	Code	Meaning	Example^a^
Simple sequence repeats	1n8	A single nucleotide repeated 8+ times in a row	GCAAAAAAAAATA
	2n5	A dinucleotide repeated 5+ times in a row	ACCACACACACATA
	3n4	Analogous to the two examples above	
	4n4		
	5n4		
	6n3		
	7n3		
	8n3		
	9n2		
	10n2		
	11n2		
Close direct repeats	4n6g12	A tetranucleotide repeated 6+ times with gaps ≤12 nt	TACCATGCTCCATTACCATAGCCAT…
	6n6g24	A 6-mer repeated 6+ times with gaps ≤24 nt	
	8n4g24	An 8-mer repeated 4+ times with gaps ≤24 nt	
	cd8g6	An 8-mer repeated within 6 nt	CTTAGGCATCACCTTAGGCA
	cd10g50	A 10-mer repeated within 50 nt	
Palindromes and inverted repeats	cp8g6	Inverted repeat of an 8-mer separated by no more than 6 nt	CTTAGGCATCACTGCCTAAG
	cp10g50	Inverted repeat of a 10-mer separated by ≤50 nt	
	pals9	9 nt inverted repeat (no separation) OR 12 nt inverted repeat allowing 1 mismatch OR 15 nt inverted repeat allowing 2 mismatches OR … (one mismatch added for every 3 nt length)	CTGGATCAGGCTAAA⋮TTCAGCCTCATCCAG
	pals9g12	Like pals9 but allowing separation up to 12 bp	
	pals12g20	Analogous to the example above	
H-DNA-related patterns	cm8g6	Mirror repeat of an 8-mer separated by ≤6 nt	CTTAGGCATCACACGGATTC
	cm10g50	Mirror repeat of a 10-mer separated by ≤50 nt	
	mirs9	9 nt mirror repeat (no separation) OR 12 nt mirror repeat allowing 1 mismatch OR 15 nt mirror repeat allowing 2 mismatches OR … (one mismatch added for every 3 nt length)	CTGGATCAGGCTAAA⋮AACTCGGACTGGGTC
	mirs9g12	Like mirs9 but allowing separation up to 12 bp	
	mirs12g20	Analogous to mirs9g12	
	R15	Run of ≥15 purines or pyrimidines	AAGGGAGGGAGGAGA
	R30	Run of ≥30 purines or pyrimidines	
	R30e3	Run of ≥30 purines or pyrimidines allowing ≤3 errors	
	R45e6	Analogous to the example above	
	R60e9		
G-DNA-related patterns	GG8g4	8 or more GG dimers separated by ≤4 nt from each other	GGAGGCTGGCGGGGCGGTGGGG
	GGG4g6	Analogous to the example above	
	GGGG4g6		
Z-DNA-related patterns	GC6	Alternating G–C, ≥6 nt length	GCGCGC
	GC8	Alternating G–C, ≥8 nt length	
	RY12	Alternating R-Y, ≥12 nt length	TGTACGTGTGCA
	RY12e1	Like RY12 but allowing 1 error	TGTACGAGTGCA
	RY18e2	Alternating R-Y, ≥18 nt length, ≤2 errors	
	RY24e3	Alternating R-Y, ≥24 nt length, ≤3 errors	
DNA bending	bend45w60	Predicted bend of ≥45° within a ≤60 bp segment	
	bend60w100	Predicted bend of ≥60° within a ≤100 bp segment	
	bend90w120	Predicted bend of ≥90° within a ≤120 bp segment	

^a^Segments matching the sequence pattern are underscored, mismatches are shaded, and symmetrical segments are separated by a vertical dashed line.

It is worthwhile to note that we are not aiming to predict accurately every site in the genome that is likely to undergo a conformational transition under favourable conditions. We are only asking how common are sequences favouring certain structural transitions in each genome and whether their frequency could be indicative of selective constraints acting on such sequences. At the same time, the extensive simulations with randomized genomes described below require that the patterns are sufficiently simple that they can be identified quickly. Given this limitation, we selected the sequence patterns to be approximately representative of the sequences known to undergo the specific structural transitions under favourable conditions.

### 2.3. Evaluation of pattern representations

Pattern Locator^36^ was used to count loci in each analysed sequence that matched the sequence pattern at hand (the observed count). The expected count of matching loci was determined as the average number of matching loci in 20 randomized sequences. The randomized sequences were generated by the ‘m1c1’ model of the Genome Randomizer software previously developed in our laboratory^37^ (available for download at http://www.cmbl.uga.edu/software.html). In this model, the analysed sequence is first divided into segments corresponding to annotated protein-coding genes and intergenic regions. For each intergenic segment, a random sequence of the same length is generated as the first-order Markov chain, thus preserving the nucleotide and dinucleotide composition of that specific intergenic segment. Analogously, a random sequence is generated for each gene as a first-order Markov chain using the codon alphabet. The final randomized genome is constructed by reassembling the randomized genes and intergenic segments in their original order. The resulting randomized sequence mimics not only the overall nucleotide, dinucleotide, and codon frequencies of the complete genome but rather of each individual gene and intergenic region. Thus, the null model incorporates the compositional heterogeneity of the genomic sequence at the scale of individual genes. Because we factor the codon usage biases, dinucleotide preferences, and differences between protein-coding and non-coding sequences into the null model, we are more likely to detect anomalous usage of sequence patterns that arises from direct selection on the sequence patterns as opposed to anomalous usage that is a simple consequence of codon or dinucleotide biases characteristic of the particular genome or its various segments. As a result, our assessments are more likely to reflect selective constraints and functional significance of the analysed sequence pattern than assessments utilizing a simpler null model.

The pattern representations are classified into nine levels, from −4 (extremely under-represented), through 0 (normally represented) to +4 (extremely over-represented) based on the *P*-value and the observed/expected ratio (Supplementary Table S8). We use the combination of the two criteria because the observed/expected ratio is independent of the sample size and measures the deviation from the null model in more absolute terms but does not directly provide assessment of statistical significance. Using *P*-value alone could emphasize small deviations in large samples (e.g. large genomes or more frequent patterns), whereas using the observed/expected ratio alone could lead to over-interpretation of results that lack statistical significance. Assigning representation categories based on both criteria is a compromise designed to avoid these potential pitfalls. The *P*-values are assessed based on an assumption that the counts of matching loci follow the Poisson distribution. This is a reasonable approximation for long sequences and patterns that occur at low frequencies.

Statistical assessments could be skewed by inclusion of observations that are not independent, such as several closely related genomes (e.g. different strains of the same species or closely related species), which are likely to feature similar levels of representations of various sequence patterns simply as a result of insufficient evolutionary divergence. To reduce the effect of dependent observations resulting from insufficient divergence, we combined the results for all genomes of the same genus into a single ‘observation’ by averaging pattern representation categories for all available genomes belonging to that genus.

## 3. Results

### 3.1. Simple sequence repeats

A strong avoidance of tandem repeats of mono-, di-, and tri-nucleotides spreads over all phyla, especially in the whole genome and protein-coding regions, while tandem repeats of longer oligomers are generally normally represented and sometimes weakly over-represented (Table 2 and Supplementary Table S9). However, tandem repeats of mono-, di-, and tri-nucleotides are generally less suppressed in the intergenic regions when compared with protein-coding regions (Supplementary Tables S10 and S11). *Trichodesmium erythraeum* and *Methanosphaera stadtmanae* are extreme examples with strongly over-represented mono-nucleotide repeats 1n8 (a single nucleotide repeated ≥8 times) in the intergenic regions (*P* < 10^−12^), but normally represented in the whole genome and extremely under-represented in the protein-coding regions. Most Chlorobi (6 species out of 10) also tend to have over-represented 1n8 pattern in the intergenic regions. Tandem repeats of longer oligonucleotides (6–11 bp) are extremely over-represented in intergenic regions as well as complete genomes of *Methanococcus voltae*, *Methanococcus aeolicus*, and *Natrialba magadii* (and to a lesser extent in several other genomes) but not in the protein-coding segments (Supplementary Tables S2–S4).

**Table 2.** Representation of sequence patterns in different phyla

Pattern name	Pattern code	AlPr	BePr	GaPr	DePr	EpPr	Firm	Acti	Cyan	Bact	Chlb	Chlf	Dein	Fuso	Chla	Spir	Acid	Verr	Defe	Plan	Aqui	Ther	Eury	Cren
		58	39	76	23	11	61	63	12	34	5	8	6	5	6	4	4	4	4	4	8	5	44	16
Simple sequence repeats	1n8	−4.00	−3.00	−4.00	−4.00	−4.00	−4.00	−4.00	−4.00	−4.00	−1.67	−4.00	−4.00	−4.00	−3.79	−3.75	−3.00	−3.00	−4.00	−3.50	−4.00	−4.00	−4.00	−4.00
	2n5	−2.21	−2.00	−3.00	−3.00	−4.00	−4.00	−3.00	−3.63	−4.00	−3.00	−3.00	−2.60	−4.00	−4.00	−3.50	−2.00	−2.50	−4.00	−2.00	−4.00	−4.00	−3.00	−3.00
	3n4	−0.78	−2.00	−2.09	−1.00	−3.00	−3.00	−2.00	−1.50	−2.67	−2.00	−1.50	−1.50	−4.00	−3.00	−3.17	−0.25	−1.00	−3.50	−1.00	−3.50	−4.00	−3.00	−3.00
	4n4	0.00	0.00	0.00	0.00	0.00	0.00	0.00	0.00	0.00	0.00	0.00	0.00	−1.00	0.00	0.00	0.00	0.00	0.00	0.00	0.00	0.00	0.00	0.00
	5n4	0.00	0.00	0.00	0.00	0.00	0.00	0.00	0.00	0.00	0.00	0.00	0.00	0.00	0.00	0.07	0.00	0.00	0.00	0.00	0.00	0.00	0.00	0.00
	6n3	0.00	0.00	0.00	0.00	0.00	0.00	0.00	0.00	0.00	0.00	0.00	0.00	0.00	0.00	0.07	0.00	0.00	0.00	0.00	0.00	0.00	0.00	0.00
	7n3	0.00	0.00	0.00	0.00	0.00	0.00	0.00	0.00	0.75	0.00	1.00	0.00	0.00	0.00	0.00	0.00	0.50	1.00	2.00	0.00	0.00	0.00	0.00
	8n3	0.00	0.00	0.00	0.00	0.00	0.00	0.00	0.00	0.00	0.00	0.00	0.00	0.00	0.00	0.50	0.00	0.00	0.50	0.00	0.00	0.00	0.00	0.00
	9n2	0.00	0.00	0.00	0.00	0.00	0.00	0.00	0.00	0.00	0.00	0.00	0.00	0.00	0.00	−0.36	0.00	0.00	0.00	0.00	0.00	0.00	0.00	0.00
	10n2	0.00	0.00	0.00	0.00	0.00	0.00	0.00	0.00	0.00	0.00	0.00	0.00	0.00	0.00	0.17	0.00	0.00	0.00	0.00	0.00	1.00	0.00	0.00
	11n2	0.00	0.00	0.00	0.00	0.00	0.00	0.00	0.59	0.00	0.00	0.00	0.00	0.00	0.00	0.00	0.00	0.00	0.00	0.00	0.00	0.00	0.00	0.00
Close direct repeats	4n6g12	0.00	0.00	0.00	0.00	0.00	0.00	1.00	0.00	0.00	0.00	0.00	0.00	0.00	0.00	0.13	0.00	0.00	0.00	1.25	0.00	0.00	0.00	0.00
	6n6g24	0.42	0.25	0.88	1.00	0.00	0.30	2.00	1.50	1.00	1.00	1.00	0.00	0.00	0.00	0.98	1.50	0.00	1.50	1.25	0.00	0.00	0.10	0.00
	8n4g24	0.50	1.00	1.14	2.00	0.00	1.00	3.00	3.00	1.50	2.00	1.00	0.00	0.00	0.00	1.25	2.25	0.50	1.50	2.75	0.00	0.00	1.00	0.00
	cd8g6	0.00	0.00	0.00	1.00	1.00	0.00	2.00	1.92	0.00	0.00	0.75	1.20	0.00	0.00	0.02	0.00	0.00	0.00	0.00	0.00	1.00	0.60	1.00
	cd10g50	0.00	0.00	0.00	0.00	0.00	0.00	1.00	0.60	0.00	0.00	0.00	0.00	0.00	−0.19	0.15	0.00	0.00	−1.00	1.00	0.00	0.00	0.00	0.00
Palindromes and inverted repeats	cp8g6	3.00	3.00	4.00	3.50	3.00	4.00	3.00	1.81	3.00	2.00	4.00	2.63	3.00	4.00	2.86	3.50	4.00	3.00	1.50	2.00	4.00	2.00	2.00
	cp10g50	3.00	4.00	4.00	4.00	3.83	4.00	4.00	3.54	4.00	4.00	4.00	3.75	4.00	4.00	3.69	3.50	2.00	3.00	3.50	1.00	4.00	1.00	1.00
	pals9	1.09	2.00	3.35	3.00	2.00	4.00	4.00	2.00	4.00	3.00	3.50	3.00	3.00	3.96	2.17	1.50	2.00	1.50	1.00	0.00	3.50	0.10	0.00
	pals9g12	3.66	4.00	4.00	4.00	3.72	4.00	4.00	3.71	4.00	4.00	4.00	3.75	4.00	4.00	3.77	4.00	4.00	3.00	4.00	1.00	4.00	2.00	1.00
	pals12g20	4.00	4.00	4.00	4.00	4.00	4.00	4.00	4.00	4.00	4.00	4.00	4.00	4.00	4.00	3.77	4.00	4.00	3.00	4.00	0.00	4.00	1.00	0.22
H-DNA-related patterns	cm8g6	0.00	0.00	0.00	0.00	0.00	0.00	0.00	0.00	0.00	0.00	0.00	0.00	0.00	0.00	0.34	0.00	0.00	0.00	0.00	0.00	0.00	0.00	0.00
	cm10g50	0.00	0.00	0.00	0.00	0.00	0.00	0.00	0.00	0.00	0.00	0.00	0.00	0.00	0.00	0.34	0.25	0.00	0.00	0.00	0.00	0.00	0.00	0.00
	mirs9	0.00	0.00	0.00	0.00	0.00	0.00	0.00	0.00	0.00	0.00	0.00	0.00	0.00	0.00	0.00	0.00	0.00	0.00	0.00	0.00	0.00	0.00	0.00
	mirs9g12	0.00	0.00	0.00	0.00	0.00	0.00	0.25	0.17	0.00	0.00	0.34	0.00	0.00	0.00	0.25	0.50	0.00	0.00	0.25	0.00	0.00	0.00	0.00
	mirs12g20	0.00	0.00	0.00	0.00	0.00	0.00	0.00	0.09	0.00	0.00	0.00	0.00	0.00	0.00	0.13	0.00	0.50	0.00	0.25	0.00	0.00	0.00	0.00
	R15	−0.20	0.00	0.00	0.00	−1.00	−0.20	0.00	−1.07	0.00	0.00	0.25	−1.00	−4.00	0.00	−0.90	−0.50	1.00	−3.00	0.00	−2.00	−3.00	−1.00	−1.00
	R30	0.00	0.00	0.00	0.00	0.00	0.00	0.00	0.00	0.00	0.00	0.00	0.00	0.00	0.00	0.38	0.00	0.00	0.00	0.00	0.00	0.00	0.00	0.00
	R30e3	0.00	0.00	0.00	0.00	0.00	0.00	0.00	0.00	0.00	0.00	0.00	0.00	−2.00	−0.25	−0.13	0.00	0.50	−1.00	0.00	−0.50	−1.50	0.00	0.00
	R45e6	0.00	0.00	0.00	0.00	0.00	0.00	0.00	0.00	0.00	0.00	0.00	0.00	−2.00	0.00	0.15	0.00	0.50	0.00	0.50	−0.50	0.00	0.00	0.00
	R60e9	0.00	0.00	0.00	0.00	0.00	0.00	0.00	0.00	0.00	0.00	0.00	0.00	0.00	0.00	0.07	0.00	0.00	0.00	0.50	0.00	0.00	0.00	0.00
G-DNA-related patterns	GG8g4	1.00	1.33	0.00	0.00	0.00	0.00	1.00	1.00	0.00	0.00	0.00	2.70	0.00	0.00	0.54	1.00	0.00	0.00	1.50	0.00	0.00	0.00	0.00
	GGG4g6	0.00	0.00	0.00	0.00	0.00	−0.63	0.00	0.00	0.00	0.00	−0.31	1.00	0.00	0.00	−0.25	0.00	0.00	0.00	0.00	−0.50	0.00	−0.42	0.00
	GGGG4g6	0.00	0.00	0.00	0.00	0.00	0.00	0.00	0.00	0.00	0.00	0.17	0.00	0.00	0.00	0.00	0.00	0.00	0.00	0.00	0.00	0.00	0.00	0.00
Z-DNA-related patterns	GC6	−1.00	−1.00	−2.00	−2.00	−1.00	−1.00	−2.00	−2.00	−1.00	−4.00	−2.00	−1.20	0.00	0.00	−0.77	−1.50	−1.00	−0.50	−2.75	−0.25	0.00	−2.00	−1.00
	GC8	−0.50	−1.00	−1.00	−1.00	0.00	0.00	−3.00	−0.25	0.00	−2.00	−1.34	−1.25	0.00	0.00	0.00	−0.50	0.00	0.00	−3.00	0.00	0.00	0.00	0.00
	RY12	−2.50	−2.00	−3.00	−1.00	−0.89	−0.33	−2.00	−2.00	−0.35	−2.00	−3.25	−0.25	0.00	0.42	−0.09	−0.75	0.00	−1.00	−3.00	0.00	0.00	−0.72	0.00
	RY12e1	−2.00	−1.40	−3.00	−1.00	−1.00	−0.25	−2.00	−3.00	0.00	−3.00	−2.25	0.00	0.00	0.84	0.05	−0.75	0.00	−1.00	−3.00	0.50	0.00	−0.64	−0.98
	RY18e2	−2.65	−2.00	−2.53	−1.00	0.00	0.00	−2.00	−2.00	0.00	−2.00	−3.00	−1.00	0.00	0.75	0.00	−0.75	0.00	0.00	−3.50	0.00	0.00	0.00	0.00
	RY24e3	−1.03	−1.50	−1.00	0.00	0.00	0.00	−1.00	−1.00	0.00	−1.00	−1.25	0.00	0.00	0.00	−0.17	−1.00	0.00	0.00	−1.00	0.00	0.00	0.00	0.00
DNA bending	bend45w60	0.28	0.00	0.07	1.00	3.00	2.00	0.00	1.50	2.00	1.00	0.17	0.45	2.00	1.50	0.50	0.00	0.00	2.50	0.00	2.00	2.00	2.00	0.00
	bend60w100	0.00	0.00	0.00	0.50	2.97	2.00	0.00	1.00	2.00	0.00	0.00	0.00	2.00	0.75	0.25	0.00	0.00	3.00	0.00	2.25	3.00	2.00	0.00
	bend90w120	0.00	0.00	0.00	0.00	3.00	1.00	0.00	1.00	1.00	0.00	0.00	0.00	3.00	0.00	0.20	0.00	0.00	3.50	0.00	2.00	3.00	1.00	0.00

Numbers in the table refer to the medians of pattern representation among all genera of the corresponding phylum. The pattern representations were categorized into nine categories from −4 (extremely under-represented), through 0 (normally represented), to +4 (extremely over-represented). See Materials and methods for details. A colour version of this table is presented in Supplementary Materials as Table S8. Codes in the second column refer to specific sequence patterns (see Table 1). Columns represent different phyla abbreviated as follows: AlPr, α-proteobacteria; BePr, β-proteobacteria; GaPr, γ-proteobacteria; DePr, δ-proteobacteria; EpPr, ɛ-proteobacteria; Firm, Firmicutes; Acti, Actinobacteria; Cyan, Cyanobacteria; Bact, Bacteroidetes; Chlb, Chlorobi; Chlf, Chloroflexi; Dein, Deinococcus-Thermus; Fuso, Fusobacteria; Chla, Chlamydiae; Spir, Spirochaetes; Acid, Acidobacteria; Verr, Verrucomicrobia; Defe, Deferribacteres; Plan, Planctomycetes; Aqui, Aquificales; Ther, Thermotogae; Eury, Euryarchaeota; Cren, Crenarchaeota. Numbers in the second row indicate the number of genera available for each phylum. Only phyla represented by three or more genera are shown.

Mono- and dinucleotide repeats are about equally suppressed in psychrophiles, mesophiles, thermophiles, and hyperthermophiles (Table 3 and Supplementary Table S12). However, the suppression of trinucleotide tandem repeats is more pronounced in thermophiles and hyperthermophiles than in mesophiles and psychrophiles (significant at *P* < 10^−5^; the *P*-value is based on Fisher's exact test for 2-by-2 contingency tables). Oxygen requirement appears to have no relationship to tandem repeat representations.

**Table 3.** Representation of sequence patterns in different OGT and oxygen requirement classes

Pattern name	Pattern code	Psychrophile	Mesophile	Thermophile	Hyperthermophile	Anaerobe	Aerobe	Facultative	Microaerophile
		18	382	72	26	159	202	95	9
Simple sequence repeats	1n8	−3.83	−3.54	−3.89	−4.00	−3.73	−3.61	−3.65	−3.67
	2n5	−3.15	−2.89	−3.40	−3.45	−3.30	−2.74	−3.02	−3.11
	3n4	−1.93	−1.92	−2.83	−3.09	−2.56	−1.75	−2.15	−2.33
	4n4	−0.06	0.01	−0.06	−0.08	−0.04	0.01	0.00	−0.10
	5n4	0.00	0.07	0.00	0.00	0.02	0.10	0.01	0.11
	6n3	0.20	0.19	0.06	−0.03	0.05	0.25	0.08	0.31
	7n3	1.27	0.48	0.45	0.00	0.42	0.59	0.35	0.89
	8n3	0.56	0.30	0.12	0.01	0.25	0.27	0.20	0.33
	9n2	−0.08	0.09	0.22	0.04	0.09	0.19	−0.11	−0.11
	10n2	0.09	0.25	0.25	0.28	0.27	0.24	0.16	0.00
	11n2	0.21	0.32	0.30	0.40	0.42	0.26	0.24	0.03
Close direct repeats	4n6g12	0.45	0.58	0.48	0.82	0.55	0.73	0.31	0.50
	6n6g24	1.35	1.16	0.71	0.34	1.00	1.21	0.81	1.39
	8n4g24	2.02	1.61	0.80	0.28	1.27	1.69	1.18	1.13
	cd8g6	0.39	0.73	0.80	1.20	0.87	0.82	0.41	0.70
	cd10g50	0.46	0.41	0.05	−0.07	0.25	0.60	0.01	0.07
Palindromes and inverted repeats	cp8g6	3.57	2.78	2.81	1.99	2.98	2.39	3.21	3.22
	cp10g50	3.56	3.26	2.98	1.64	3.15	3.03	3.45	3.20
	pals9	3.39	2.58	2.13	0.71	2.47	2.27	2.89	2.47
	pals9g12	3.83	3.46	3.05	1.79	3.33	3.22	3.55	3.64
	pals12g20	3.72	3.58	2.97	1.17	3.16	3.47	3.62	3.00
H-DNA-related patterns	cm8g6	0.02	0.20	0.21	0.33	0.24	0.24	0.15	0.04
	cm10g50	0.02	0.16	0.10	0.15	0.16	0.18	0.10	0.00
	mirs9	0.00	0.13	0.19	0.17	0.14	0.18	0.08	−0.02
	mirs9g12	0.15	0.30	0.35	0.44	0.33	0.38	0.20	0.00
	mirs12g20	0.15	0.27	0.28	0.20	0.26	0.33	0.16	0.22
	R15	−0.47	−0.52	−0.96	−1.77	−0.91	−0.55	−0.34	−1.78
	R30	0.03	0.07	0.08	0.11	0.10	0.04	0.07	0.00
	R30e3	0.08	−0.01	−0.18	−0.66	−0.23	0.00	0.14	−0.66
	R45e6	0.17	0.08	−0.03	−0.32	−0.06	0.10	0.15	−0.02
	R60e9	0.15	0.13	0.11	−0.03	0.14	0.10	0.12	0.00
G-DNA-related patterns	GG8g4	0.22	0.72	0.24	0.14	0.20	1.03	0.48	0.44
	GGG4g6	−0.08	−0.26	−0.78	−0.65	−0.67	−0.18	−0.26	−0.22
	GGGG4g6	0.00	0.10	−0.06	−0.12	0.01	0.11	0.02	0.11
Z-DNA-related patterns	GC6	−1.37	−1.50	−1.35	−0.95	−1.41	−1.38	−1.71	−1.42
	GC8	−0.75	−1.10	−0.84	−0.18	−0.47	−1.42	−1.13	−0.37
	RY12	−1.79	−1.58	−0.88	−0.16	−0.78	−1.73	−2.03	−0.99
	RY12e1	−1.81	−1.40	−0.71	−0.31	−0.85	−1.43	−1.93	−0.44
	RY18e2	−1.66	−1.45	−0.72	−0.04	−0.58	−1.60	−2.07	−0.37
	RY24e3	−0.60	−0.78	−0.25	0.05	−0.30	−0.79	−1.12	−0.44
DNA Bending	bend45w60	0.60	0.93	1.51	1.11	1.57	0.59	0.85	1.70
	bend60w100	0.32	0.79	1.40	1.17	1.42	0.50	0.74	1.48
	bend90w120	0.28	0.58	1.23	0.94	1.11	0.34	0.66	1.44

Numbers in the table refer to the average significance category for all genera within each class of organisms. Numbers below the class description indicate numbers of available genera of each class. Anaerobe includes both obligate anaerobes and anaerobes; Aerobe includes both obligate aerobes and aerobes. See Supplementary Table S5 for coloured version of this table.

### 3.2. Close direct repeats

In general, close direct repeats are normally represented or slightly over-represented in complete genomes of most prokaryotes and in the intergenic regions, and they are mostly normally represented in genes (Supplementary Tables S9–S11). The cd10g50 repeats (two copies of the same 10-mer separated by ≤50 nucleotides) exhibit the strongest trends among the investigated direct repeat structures. They are mostly over-represented in the intergenic regions, weakly under-represented in protein-coding regions, and normally represented in the whole genome. It is interesting to note the contrast in representations of close pairs of 10 bp repeats (cd10g50) and multiple close copies of shorter oligonucleotides (4n6g12, 6n6g24, 8n4g24). While the former are often strongly over-represented in intergenic regions and normally represented to weakly under-represented in genes, the latter are normally represented or moderately over-represented in both genes and intergenic regions (Supplementary Tables S10 and S11).

**Figure 1.**
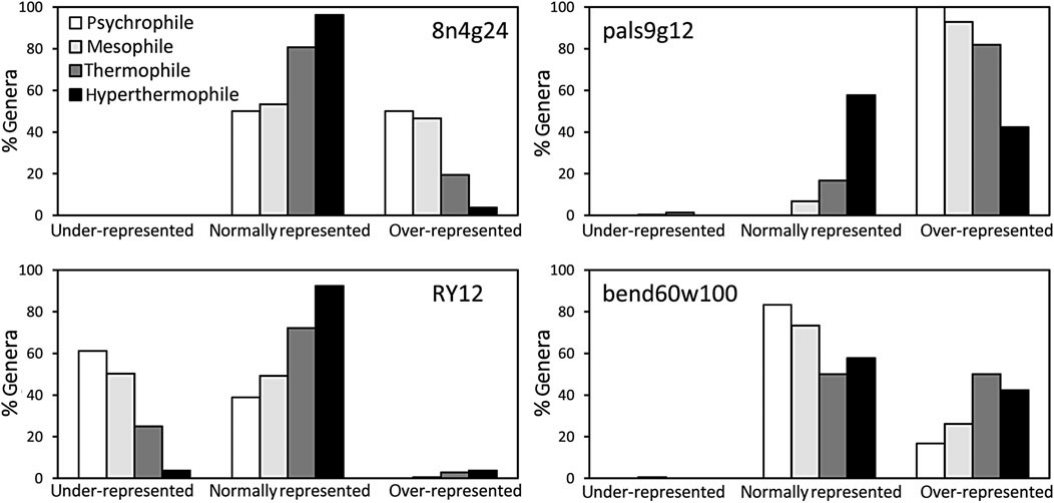
Comparison of representations of selected patterns in different OGT classes. Bars show the percentage of species in each OGT class which have the given pattern under-represented, normally represented, or over-represented. The pattern is considered over-represented if the *P*-value is <10^−4^ and observed to expected ratio >1.10 (representation level 2 or higher) for majority of the complete genomes available for that genera, it is deemed under-represented if the *P*-value is <10^−4^ and observed to expected ratio <0.91, and normally represented otherwise. See Materials and methods and Supplementary Table S8. The four patterns for which the data are shown are representative of close repeat structures (8n4g24, top left), palindromes and close inverted repeats (pals9g12, top right), potential Z-DNA-promoting patterns (RY12, bottom left), and DNA bending pattrens (bend60w100, bottom right). See Table 1 for description of the pattern codes.

Representations of close direct repeats exhibit clear negative trends with increasing OGT (Table 3 and Supplementary Table S12). For example, clustered repeats 8n4g24 (at least four copies of the same octamer separated by gaps of no more than 24 bp) are over-represented in ∼50% of psychrophiles and mesophiles but in few thermophiles and virtually no hyperthermophiles (Fig. 1; the difference is significant at *P* < 10^−6^). A significant decrease in pattern representations with increasing OGT is seen also for patterns 6n6g24 and8n4g24, indicating a general trend of lower local repetitiveness in thermophiles and hyperthermophiles. There is no clear relationship between the representation of close direct repeats and oxygen requirement.

### 3.3. Palindromes and inverted repeats

Palindromes and inverted repeats are strongly over-represented in the complete genomes across almost all bacterial phyla but less so in archaea and Aquificales (Table 2 and Supplementary Table S9). There are few organisms that strongly avoid palindromes in protein-coding regions and the complete genomes, including cyanobacteria *Synechosystis* and *Thermosynechococcus* (Supplementary Tables S2 and S3). Representations of palindromes and close inverted repeats in protein-coding sequences vary significantly among different genera, from extremely over-represented (e.g. *Cyanobacterium*, *Thermoanaerobacter*, *Allivibrio*, *Azobacteroides*) to strongly suppressed (e.g. *Rhodomicrobium*, *Phenylobacterium*, *Jannaschia*, *Clavibacter*). Many α-proteobacteria exhibit some level of palindrome suppression. *Clavibacter* is an outlier among Actinobacteria, which generally tend to have palindromes normally represented and in some cases over-represented (Supplementary Table S3).

A comparison between genes and intergenic regions shows that inverted repeats are almost invariably over-represented in the intergenic regions and generally normally represented in the protein-coding regions (Supplementary Tables S3 and S4). The high concentration of palindromes and inverted repeats in the intergenic region is not necessarily surprising because of their function in transcription termination and as regulatory elements, which are generally located outside the protein-coding segments of genes.

There is a general trend of decreasing representations of palindromes and inverted repeats with increasing OGT (Fig. 1, Table 3, and Supplementary Table S12). In contrast, there is only a weak or no relationship between representations of palindromes and oxygen requirement.

### 3.4. Oligopurine/oligopyrimidine runs and triplex DNA-promoting patterns

H-DNA triplexes form under favourable conditions in oligopurine/oligopyrimidine sequences with a mirror symmetry,^24,26^ whereas some oligopurine/oligopyrimidine segments can also promote formation of A-like DNA.^2^ Mirror repeats and extended oligopurine/oligopyrimidine stretches are generally normally represented in complete genomes of most phyla (Table 2 and Supplementary Table S9), with moderate over-representations in some Cyanobacteria (*Anabaena, Nostoc*, *Microcystis, Trichodesmium*), Chloroflexi (*Chloroflexus* and *Roseiflexus*), Planctomycetes (*Isosphaera*), and Verrucomicrobia (*Methylacidiphilum*) (Supplementary Table S2). However, oligopurine and oligopyrimidine stretches, mainly patterns R15 and R30e3, are suppressed in complete genomes of some phyla. Separate analysis of protein-coding and non-coding regions shows that oligopurine/oligopyrimidine stretches tend to be normally represented in intergenic regions and that anomalous representations of these patterns in some phyla arise from biases in protein-coding segments (Supplementary Tables S10 and S11).

Suppression of oligopurine/oligopyrimidine stretches, especially R15, is most common among hyperthermophiles, whereas mesophiles and psychrophiles exhibit normal representations of these patterns (Table 3 and Supplementary Table S12). The R15 pattern is also more likely to be under-represented in anaerobes than in aerobes (Table 3, Supplementary Table S12, and Supplementary Fig. S1). On the other hand, mirror repeats cm10g50 and mirs12g20 are more likely to be over-represented in the coding regions of anaerobes than aerobes (Supplementary Table S13 and Supplementary Fig. S2).

### 3.5. Guanine-rich patterns and G-DNA-promoting sequences

Formation of intrastrand G-DNA quadruplex is promoted by clustered short runs of guanine.^17–19^ We investigated several forms of such G-rich clusters (G-patterns; Table 1), among which the GGG4g6 pattern (four G-triplets separated by gaps of no more than six nucleotides) represents best the sequences known to form G-quadruplexes.^2,38,39^ In general, the G-patterns are normally represented in prokaryotic genomes (Table 2 and Supplementary Table S9). GG dimer clusters (GG8g4, eight or more GG dimers separated by ≤4 nucleotides from each other) tend to be moderately over-represented in protein-coding regions of α-, β-, and δ-proteobacteria, Actinobacteria, Cyanobacteria, Deincoccus-Thermus group, and Planctomycetes (especially for *Isosphaera*) (Supplementary Table S10). The other guanine patterns are generally normally represented with most notable exceptions among Cyanobacteria where species of *Microcystis*, *Anabaena*, and *Nostoc* feature extreme over-representation of all forms of G-patterns (Supplementary Table S2). The planctomycete *Isosphaera pallida* also has G-patterns strongly over-represented. With these few exceptions, the GGG4g6 pattern, which is most directly related to G-DNA formation, is mostly normally represented or slightly under-represented, suggesting that if G-DNA-promoting sequences have significant physiological roles in bacteria, such roles are probably limited to only a few species or genera.

Most organisms with over-represented GG8g4 pattern are aerobes, whereas virtually no anaerobes have excess of GG8g4 (Supplementary Fig. S1). However, over-represented GG8g4 pattern could also reflect excess of glycine-rich segments (encoded by the GGN codons) or proline-rich segments (encoded by CCN with GG dinucleotides in the complementary strand) in proteins, whereas the GGG4g6 does not exhibit a relationship with oxygen requirement. There is no apparent relationship between G-pattern representations and the OGT (Table 3 and Supplementary Table S12).

### 3.6. Z-DNA-promoting patterns

The left-handed Z-DNA conformation is most commonly adopted by runs of alternating G–C and more generally by alternating purine–pyrimidine (RY) patterns. The RY patterns are often under-represented but to different extent in different phyla—most strongly in α-, β-, and γ-proteobacteria, Actinobacteria, Cyanobacteria, Chlorobi, Chloroflexi, and Planctomycetes (Table 2 and Supplementary Table S9). In contrast, the RY patterns are normally represented in Fusobacteria, Aquificales, and Thermotogae and even slightly over-represented in Chlamydiae. In general, the suppression of RY patterns is stronger in protein-coding regions, although several phyla exhibit significant RY pattern suppression in intergenic regions as well (Supplementary Tables S10 and S11). Interestingly, *Treponema pallidum* and *Treponema paraluiscuniculi* exhibit a strong over-representation of all forms of RY patterns, whereas other spirochaetes, including other species of *Treponema*, have RY patterns normally represented or weakly under-represented. Similar extreme over-representation of RY patterns applies to *Helicobacter felis* and to a lesser extent to *Helicobacter bizzozeronii* but not to other *Helicobacter* species. The archaeon *Thermofilum pendens* also exhibits a strong over-representation of RY patterns and several other genomes show a weaker RY-pattern over-representation (Supplementary Table S2).

We used Pattern Locator and associated software tools^36,40^ (http://www.cmbl.uga.edu/software/patloc.html) to investigate in detail the distribution of RY patterns in several specific genomes, including the above-mentioned genomes with over-represented RY patterns. We did not find any significant anomalies in the distribution of the RY patterns with respect to the origin or terminus of replication, with respect to the 3′ ends of genes (stop codons), or any strong association with a particular class of genes. However, we noted increased numbers of RY patterns overlapping with start codons, such that the ATG/GTG start codons are embedded in RY patterns which sometimes extend several codons deep into the protein-coding region (Supplementary Fig. S3). This tendency appears to be widespread among prokaryotic genomes. However, comparison between ATG and GTG triplets that function as start codons and those that are not translation start sites shows no significant difference in fractions of ATG/GTG triplets overlapping with extended RY patterns between the two groups (Supplementary Table S18). This suggests that the increased number of RY patterns overlapping with translation start sites is not directly related to the translation but rather a simple consequence of ATG and GTG themselves having the form of a short RY pattern (RYR), thus increasing the likelihood of finding a longer RY pattern at the same site.

Representations of RY patterns tend to increase with increasing OGT (Table 3 and Supplementary Table S12). For example, the pattern RY12 is under-represented in 60% of psychrophiles, 50% of mesophiles, ∼25% of thermophiles but only a single hyperthermophilic genus, *Thermaerobacter* (Fig. 1; significant at *P* < 10^−6^).

Among all analysed sequence patterns, the RY patterns exhibit the most notable trend with respect to the level of oxygen requirement (Table 3 and Supplementary Table S12). Interestingly, facultative species exhibit the strongest suppression of RY patterns followed by aerobes, whereas RY pattern representations in anaerobes and microaerophilic organisms are close to normal (see Supplementary Fig. S1c for pattern RY12). The same trend was observed when the comparisons between aerobes and anaerobes were restricted to mesophiles, or bacteria (Supplementary Tables S15 and S17), indicating that the relationship between RY patterns and oxygen requirement cannot be attributed to increased number of anaerobes among thermophiles or different representations of anaerobes and aerobes among bacteria and archaea.

### 3.7. Patterns contributing to DNA curvature

Sequence patterns related to DNA curvature (the bend-patterns) range generally from normal to over-represented. The over-representation is most pronounced in ɛ-proteobacteria, Fusobacteria, Deferribacteres, and Thermotogae (Table 2 and Supplementary Table S8). This trend applies to both protein-coding and intergenic regions, although differences among phyla are more prominent in protein-coding regions (Supplementary Tables S9 and S11). At the opposite extreme, *Mycoplasma haemofelis*, *Mycoplasma penetrans*, *Cytophaga hutchinsonii*, and *Pelagibacter* sp. show moderate to strong suppression of DNA bends in their genomes (Supplementary Table S2). The genus *Mycoplasma* is particularly interesting, featuring species with extreme over-representation of DNA bends as well as strong under-representation. This is consistent with our previous report that various genome properties among *Mycoplasma* vary more than in other genera.^37^ In addition, *C. hutchinsonii* and *Pelagibacter* sp. feature extreme suppression of bend-patterns in protein-coding regions but not in intergenic regions (Supplementary Tables S3 and S4).

With respect to OGT, intrinsic DNA bends tend to be more over-represented in hyperthermophiles and thermophiles than in mesophiles and psychrophiles (Table 3 and Fig. 1). Surprisingly, the number of genera with over-represented bend-patterns in protein-coding segments increases with increasing OGT in protein-coding regions but decreases for bend-patterns in non-coding regions (Supplementary Fig. S4). The same trend is shown in Supplementary Fig. S5, where genomes of thermophiles and hyperthermophiles tend to have more protein-coding bends than intergenic bends, whereas the opposite is true for most psychrophiles. Statistical significance of this trend has been confirmed by the Mann–Whitney *U*-test (Supplementary Table S19).

With respect to oxygen requirement, representation of DNA bending patterns tends to decrease with the increasing level of oxygen (Table 3 and Supplementary Table S12). Specifically, the bend-patterns tend to be over-represented in anaerobes and microaerophiles but not in aerobes. For example, the pattern bend60w100 is over-represented in half of the anaerobes, but <20% of aerobes (Supplementary Fig. 1; significant at *P* < 10^−6^). This trend holds when the data are restricted to mesophiles or thermophiles, suggesting that the trend with respect to oxygen requirement is independent of the trend with respect to OGT (Supplementary Tables S15 and S16).

## 4. Discussion

### 4.1. Comparisons with previous work

The work presented here differs from similar earlier surveys in scope (both the number of genomes used in the analysis and the number of different types of sequence patterns investigated) as well as methodology. The most important methodological difference is in the null model used to assess whether a sequence pattern is anomalously represented in a given genome. Our null model takes into account the nearest-neighbour biases and codon usage propensities of all individual genes and intergenic regions, which likely have separate underlying causes not related to potential functional significance of the investigated sequence patterns. In terms of our results, one significant difference relative to earlier work concerns representations of alternating purine–pyrimidine patterns. Bohlin *et al.*^3^ reported that alternating RY patterns were generally suppressed across different phyla except β-proteobactria, where they were mostly over-represented, with a particularly strong surplus of RY patterns in *Burkholderia*. These authors used the i.i.d. model (independently drawn and independently distributed letters) as a benchmark. Using our more realistic null model, we found that β-proteobactria including *Burkholderia* species suppress the RY patterns to the same extent as other bacteria (Table 2 and Supplementary Tables S2 and S9). We therefore conclude that the increased amount of RY patterns in β-proteobactria arises from two opposite evolutionary constraints: biased codon and dinucleotide usages that favour RY-patterns and are specific for β-proteobactria, which are partially offset by suppression of long RY-patterns, which is rather universal among bacterial genomes. Similarly, the over-representation of oligopurine/oligopyrimidine stretches reported by Bohlin *et al.* for some phyla arises from the biases at the level of dinucleotide and codon usages, as we found the oligopurine/oligopyrimidine stretches normally represented or under-represented in all phyla (Table 2 and Supplementary Table S9).

For SSRs, our results are consistent with previous works including our own survey of SSRs in prokaryotic genomes.^7^ In particular, our present data confirm that SSR comprised tandem repeats of very short units (mono-, di-, and trinucleotides; patterns 1n8, 2n5, and 3n4) tend to be strongly suppressed in prokaryotic genomes, while repeats of longer units (tetranucleotides through 11-mers) tend to be normally represented or weakly over-represented (Table 2 and Supplementary Table S9). This strong difference points to likely functional difference between tandem repeats of very short (1–3 bp) and longer (4–11 bp) units. Our present results differ from the earlier data in that we previously included repeats of tetranucleotides in the same group as mono-, di, and trinucleotides,^7^ while the present results indicate that tetranucleotide SSRs may be more accurately included in the same group as SSRs composed of pentanucleotides and longer units, which are generally not suppressed. This result suggests that tandem repeats of tetranucleotides and longer units may not have the same harmful effects as repeats of mono-, di-, and trinucleotides. The difference between tandem repeats of very short units (1–3 bp) and longer units (≥4 bp) could also arise from properties of the methyl-directed repair pathway, which is very efficient in repairing heterologous loops of 1–3 bp, but its efficiency dramatically drops as the length of the loop increases.^41,42^

Ladoukakis and Eyre-Walker^43^ reported a small but significant excess of short inverted repeats (6–9 bp) in protein-coding sequences and proffered that the inverted repeats could arise by sequence-directed mutagenesis where an imperfect palindrome or inverted repeat converts to a perfect one due to template switching during replication.^15^ Their result is consistent with that of Katz and Burge,^44^ who found that native mRNA sequences in many bacterial genomes possess a significantly higher potential to form stable RNA secondary structures than random sequences preserving the codon usage, dinucleotide frequencies, and the protein sequences encoded by the native mRNAs. Katz and Burge proposed that the excess of base pairing in mRNA molecules is due to selection for stable mRNAs. We analysed occurrences of slightly larger palindromes than Ladoukakis and Eyre-Walker but with a similar result that palindromes and close inverted repeats are strongly over-represented in both protein-coding and non-coding regions of genomes of most prokaryotic phyla.

The excess of palindromes is weaker in the archaea and in the Aquificae than in other bacterial phyla. This result is similar to that of Katz and Burge, who reported a significant excess of mRNA secondary structures in most bacteria but few archaea, and not in *Aquifex aeolicus* (note that their study was based on a much smaller sample of genomes available at that time and *A. aeolicus* was a sole representative of Aquificae in their data set). The weaker over-representation of palindromes in archaea and Aquificae is related to general decrease in palindrome representations with increasing OGT (Fig. 1). The decrease in palindrome representations with increasing OGT might seem counterintuitive if selection for mRNA stability drives the excess of palindromes in mRNA sequences. One possible explanation is that higher temperatures prevent formation of stable mRNA structures even with an increased content of inverted repeats, thus making selection for inverted repeats moot, or perhaps higher temperature decreases the efficiency of palindrome conversion via sequence-directed mutagenesis. Yet another potential explanation for weaker over-representation of inverted repeats in thermophiles stems from the work by Paz *et al.*,^45^ who noted that thermophilic mRNA sequences have increased purine-to-pyrimidine ratios compared with mesophiles, as well as excess of short oligopurine tracts. These authors proposed that purine-loading of mRNA sequences could increase their thermal stability. The resulting purine–pyrimidine imbalance could decrease the base-pairing potential in the mRNA sequences and the purine bias could therefore contribute to lower excess of palindromes and inverted repeats in thermophiles.

### 4.2. Relationship with OGT and oxygen requirement

Extreme temperature as well as presence of oxygen (via reactive oxygen species) cause damage to DNA and require cellular mechanisms to prevent or correct the damage in order to keep the cells viable. Such protection can be enzymatic (e.g. detoxification pathways) but could also involve adaptations in DNA composition and structure. One particularly puzzling question is how thermophiles prevent their DNA from denaturing. The simple explanation that DNA of thermophile is more GC-rich and thus more stable was mostly rejected as more data became available.^46,47^ Kawashima *et al*.^48^ proffered excess of RR and YY dinucleotides over RY and YR dinucleotides as a characteristic of thermophiles but this has later been shown a poor indicator of thermophily.^47^ We were therefore interested in finding whether any of the sequence patterns we analysed could be related to differences in OGT and oxygen requirement, and possibly contribute to the protection of DNA from the effects of extreme temperature and/or oxygen damage.

One of our more surprising results concerns the relationship of representations of intrinsic DNA bends with OGT. Bolshoy and coworkers^5,49^ reported an excess of intrinsically bent DNA in intergenic regions of prokaryotic genomes, including segments containing putative promoters and transcription terminators. Notably, the amount of bent DNA in intergenic regions was significantly weaker in thermophiles than in mesophiles. In a separate work, Tolstorukov *et al*.^33^ found intrinsic DNA bends distributed widely throughout bacterial genomes, including both intergenic and protein-coding regions, and they proposed that the intrinsic bends could play a role in compaction of the bacterial nucleoid. Our present data show that predicted intrinsic DNA bends are over-represented in many phyla in both protein-coding and non-coding segments. Interestingly, the protein-coding and non-coding regions exhibit opposite trends with respect to OGT. For non-coding sequences, our results are consistent with the decrease in curved DNA in thermophiles observed by Kozobay-Avraham *et al.*^5^ However, the representation of DNA bends in protein-coding sequences tends to increase with increasing OGT.

We propose that the opposite trends in protein-coding and non-coding DNA segments reflect different roles of DNA bends. Intergenic bends are often associated with regulatory elements and could facilitate opening of the DNA double helix at the transcription initiation sites.^50,51^ Decreased amount of intrinsic DNA bends in intergenic regions of thermophiles is consistent with experiments that showed that anomalous gel mobility characteristic of intrinsically curved DNA disappears at increased temperatures.^52^ If sequence-directed intrinsic DNA bending is ineffective at high temperatures, then selection for intrinsic bends at regulatory sites could be weak or absent. It is also possible that DNA bending is less important to transcription initiation at high temperatures. Surprisingly, the excess content of intragenic bends, which are less likely to have direct regulatory functions but may contribute to establishing and maintaining the nucleoid structure, increases with increasing OGT. The latter result could possibly indicate that the intrinsic bends maintain their role in stabilizing the nucleoid structure even at high temperatures and that high OGT may require increased amount of intrinsic DNA bending to maintain adequate structural stability of the chromosome.

In addition to palindromes and close inverted repeats discussed above, some direct repeat structures also feature decreasing representations with increasing OGT (Table 3 and Fig. 1). We speculate that higher temperature might make the thermophile DNA more susceptible to illegitimate recombination, DNA polymerase slippage, or other forms of mutations facilitated by short repeats, and that reducing the amount of repeats, both direct and inverted, could be a strategy to counteract increased recombination rates. Along these lines, thermophiles were reported to have lower mutation rates than mesophiles, possibly driven by selection to maintain thermostability of the encoded proteins.^53^ Because close repeats are often sources of mutations, suppression of close repeats could be a part of the strategy to decrease overall mutation rates in thermophiles.

Alternating RY patterns exhibit trends related to both OGT and oxygen requirement and these trends are independent of each other. The relationship with oxygen requirement is particularly intriguing. Facultative organisms suppress RY patterns more strongly than both aerobes and anaerobes. The main known structural effect of RY patterns is that they can facilitate transitions to left-handed Z-DNA under favourable conditions. The B-to-Z transition in such sequences can be induced by torsional stress arising from processes that require DNA unwinding, such as transcription or replication.^28,31^ The general suppression of RY patterns in prokaryotes suggests that B-to-Z DNA transitions are generally undesirable. It is intriguing to speculate that Z-DNA could be more detrimental to facultative organisms perhaps due to interference with effective regulation of a diverse ensemble of metabolic pathways related to the ability to grow in both aerobic and anaerobic conditions. However, the specific mechanism of such interference between Z-DNA formation and transcriptional regulation is not clear.

Overall, our data indicate potential new mechanisms how genome properties adapt to particular environments. Most of these mechanisms are related to adaptations to growth at high temperatures, which appear to be accompanied by reduction in overall repetitiveness of the DNA sequence, reduced excess of palindromes, and reduced DNA curvature in regulatory regions accompanied by general increase in intrinsically curved DNA in protein-coding sequences.

## Supplementary data

Supplementary data are available at www.dnaresearch.oxfordjournals.org.

## Funding

This work is supported by the grant number DBI-0950266 from National Science Foundation.

## Supplementary Material

Supplementary Data

## References

[1] Ussery D., Soumpasis D.M., Brunak S., Staerfeldt H.H., Worning P., Krogh A. (2002). Bias of purine stretches in sequenced chromosomes. Comput. Chem..

[2] Sinden R.R. (1994). DNA Structure and Function.

[3] Bohlin J., Hardy S.P., Ussery D.W. (2009). Stretches of alternating pyrimidine/purines and purines are respectively linked with pathogenicity and growth temperature in prokaryotes. BMC Genomics.

[4] Rawal P., Kummarasetti V.B., Ravindran J. (2006). Genome-wide prediction of G4 DNA as regulatory motifs: role in *Escherichia coli* global regulation. Genome Res..

[5] Kozobay-Avraham L., Hosid S., Bolshoy A. (2006). Involvement of DNA curvature in intergenic regions of prokaryotes. Nucleic Acids Res..

[6] Mrázek J. (2010). Comparative analysis of sequence periodicity among prokaryotic genomes points to differences in nucleoid structure and a relationship to gene expression. J. Bacteriol..

[7] Mrázek J., Guo X.X., Shah A. (2007). Simple sequence repeats in prokaryotic genomes. Proc. Natl Acad. Sci. USA.

[8] Thanbichler M., Wang S.C., Shapiro L. (2005). The bacterial nucleoid: a highly organized and dynamic structure. J. Cell Biochem..

[9] Dillon S.C., Dorman C.J. (2010). Bacterial nucleoid-associated proteins, nucleoid structure and gene expression. Nat. Rev. Microbiol..

[10] Moxon E.R., Rainey P.B., Nowak M.A., Lenski R.E. (1994). Adaptive evolution of highly mutable loci in pathogenic bacteria. Curr. Biol..

[11] van der Woude M.W., Baumler A.J. (2004). Phase and antigenic variation in bacteria. Clin. Microbiol. Rev..

[12] Brázda V., Laister R.C., Jagelská E.B., Arrowsmith C. (2011). Cruciform structures are a common DNA feature important for regulating biological processes. BMC Mol. Biol..

[13] Levinson G., Gutman G.A. (1987). Slipped-strand mispairing: a major mechanism for DNA sequence evolution. Mol. Biol. Evol..

[14] Dutra B.E., Lovett S.T. (2006). Cis and trans-acting effects on a mutational hotspot involving a replication template switch. J. Mol. Biol..

[15] Lovett S.T. (2004). Encoded errors: mutations and rearrangements mediated by misalignment at repetitive DNA sequences. Mol. Microbiol..

[16] Chuzhanova N., Abeysinghe S.S., Krawczak M., Cooper D.N. (2003). Translocation and gross deletion breakpoints in human inherited disease and cancer II: potential involvement of repetitive sequence elements in secondary structure formation between DNA ends. Hum. Mutat..

[17] Burge S., Parkinson G.N., Hazel P., Todd A.K., Neidle S. (2006). Quadruplex DNA: sequence, topology and structure. Nucleic Acids Res..

[18] Vorlíčková M., Bednářová K., Kejnovská I., Kypr J. (2007). Intramolecular and intermolecular guanine quadruplexes of DNA in aqueous salt and ethanol solutions. Biopolymers.

[19] Shafer R.H., Smirnov I. (2000). Biological aspects of DNA/RNA quadruplexes. Biopolymers.

[20] Schaeffer C., Bardoni B., Mandel J.L., Ehresmann B., Ehresmann C., Moine H. (2001). The fragile X mental retardation protein binds specifically to its mRNA via a purine quartet motif. EMBO J..

[21] Pan B., Shi K., Sundaralingam M. (2006). Base-tetrad swapping results in dimerization of RNA quadruplexes: implications for formation of the i-motif RNA octaplex. Proc. Natl Acad. Sci. USA.

[22] Arthanari H., Bolton P.H. (2001). Functional and dysfunctional roles of quadruplex DNA in cells. Chem. Biol..

[23] Sundquist W.I., Heaphy S. (1993). Evidence for interstrand quadruplex formation in the dimerization of human immunodeficiency virus 1 genomic RNA. Proc. Natl Acad. Sci. USA.

[24] Belotserkovskii B.P., Veselkov A.G., Filippov S.A., Dobrynin V.N., Mirkin S.M., Frank-Kamenetskii M.D. (1990). Formation of intramolecular triplex in homopurine–homopyrimidine mirror repeats with point substitutions. Nucleic Acids Res..

[25] Rustighi A., Tessari M.A., Vascotto F., Sgarra R., Giancotti V., Manfioletti G. (2002). A polypyrimidine/polypurine tract within the Hmga2 minimal promoter: a common feature of many growth-related genes. Biochemistry.

[26] Zain R., Sun J.S. (2003). Do natural DNA triple-helical structures occur and function in vivo?. Cell Mol. Life Sci..

[27] Rich A., Nordheim A., Azorin F. (1983). Stabilization and detection of natural left-handed Z-DNA. J. Biomol. Struct. Dyn..

[28] van Holde K., Zlatanova J. (1994). Unusual DNA structures, chromatin and transcription. Bioessays.

[29] Herbert A., Rich A. (1999). Left-handed Z-DNA: structure and function. Genetica.

[30] Rich A., Zhang S. (2003). Timeline: Z-DNA: the long road to biological function. Nat. Rev. Genet..

[31] Wang G., Vasquez K.M. (2007). Z-DNA, an active element in the genome. Front Biosci..

[32] Jain A., Wang G., Vasquez K.M. (2008). DNA triple helices: biological consequences and therapeutic potential. Biochimie.

[33] Tolstorukov M.Y., Virnik K.M., Adhya S., Zhurkin V.B. (2005). A-tract clusters may facilitate DNA packaging in bacterial nucleoid. Nucleic Acids Res..

[34] Herzel H., Weiss O., Trifonov E.N. (1998). Sequence periodicity in complete genomes of archaea suggests positive supercoiling. J. Biomol. Struct. Dyn..

[35] Pagani I., Liolios K., Jansson J. (2012). The Genomes OnLine Database (GOLD) v.4: status of genomic and metagenomic projects and their associated metadata. Nucleic Acids Res..

[36] Mrázek J., Xie S. (2006). Pattern locator: a new tool for finding local sequence patterns in genomic DNA sequences. Bioinformatics.

[37] Mrázek J. (2006). Analysis of distribution indicates diverse functions of simple sequence repeats in Mycoplasma genomes. Mol. Biol. Evol..

[38] Lane A.N., Chaires J.B., Gray R.D., Trent J.O. (2008). Stability and kinetics of G-quadruplex structures. Nucleic Acids Res..

[39] Vorlíčková M., Chládková J., Kejnovská I., Fialová M., Kypr J. (2005). Guanine tetraplex topology of human telomere DNA is governed by the number of (TTAGGG) repeats. Nucleic Acids Res..

[40] Mrázek J., Xie S., Guo X., Srivastava A. (2008). AIMIE: a web-based environment for detection and interpretation of significant sequence motifs in prokaryotic genomes. Bioinformatics.

[41] Fang W., Wu J.Y., Su M.J. (1997). Methyl-directed repair of mismatched small heterologous sequences in cell extracts from Escherichia coli. J. Biol. Chem..

[42] Parker B.O., Marinus M.G. (1992). Repair of DNA heteroduplexes containing small heterologous sequences in Escherichia coli. Proc. Natl Acad. Sci. USA.

[43] Ladoukakis E.D., Eyre-Walker A. (2008). The excess of small inverted repeats in prokaryotes. J. Mol. Evol..

[44] Katz L., Burge C.B. (2003). Widespread selection for local RNA secondary structure in coding regions of bacterial genes. Genome Res..

[45] Paz A., Mester D., Baca I., Nevo E., Korol A. (2004). Adaptive role of increased frequency of polypurine tracts in mRNA sequences of thermophilic prokaryotes. Proc. Natl Acad. Sci. USA.

[46] Haney P.J., Badger J.H., Buldak G.L., Reich C.I., Woese C.R., Olsen G.J. (1999). Thermal adaptation analyzed by comparison of protein sequences from mesophilic and extremely thermophilic *Methanococcus* species. Proc. Natl Acad. Sci. USA.

[47] Suhre K., Claverie J.M. (2003). Genomic correlates of hyperthermostability, an update. J. Biol. Chem..

[48] Kawashima T., Amano N., Koike H. (2000). Archaeal adaptation to higher temperatures revealed by genomic sequence of Thermoplasma volcanium. Proc. Natl Acad. Sci. USA.

[49] Bolshoy A., Nevo E. (2000). Ecologic genomics of DNA: upstream bending in prokaryotic promoters. Genome Res..

[50] Olivares-Zavaleta N., Jauregui R., Merino E. (2006). Genome analysis of Escherichia coli promoter sequences evidences that DNA static curvature plays a more important role in gene transcription than has previously been anticipated. Genomics.

[51] Jauregui R., Abreu-Goodger C., Moreno-Hagelsieb G., Collado-Vides J., Merino E. (2003). Conservation of DNA curvature signals in regulatory regions of prokaryotic genes. Nucleic Acids Res..

[52] Ussery D.W., Higgins C.F., Bolshoy A. (1999). Environmental influences on DNA curvature. J. Biomol. Struct. Dyn..

[53] Drake J.W. (2009). Avoiding dangerous missense: thermophiles display especially low mutation rates. PLoS Genet..

