# Supplementary material for: Assessing Diversity of DNA Structure-Related Sequence Features in Prokaryotic Genomes
Source: DNA Res. 2014 Jan 9;21(3):285–97. doi: 10.1093/dnares/dst057 (PMC4060949; doi:10.1093/dnares/dst057)
Supplement: Supplementary Data [file supp_21_3_285__index.html]

Assessing Diversity of DNA Structure-Related Sequence Features in Prokaryotic Genomes — Supplementary Data 

# Assessing Diversity of DNA Structure-Related Sequence Features in Prokaryotic Genomes

## Supplementary Data

Supplementary Data

**Files in this Data Supplement:**

- Supplementary Data - Pdf file
- Supplementary Table 8 to 19 - pdf file
- Supplementary Table 1 - xlsx file
- Supplementary Table 2 - xlsx file
- Supplementary Table 3 - xlsx file
- Supplementary Table 4 - xlsx file
- Supplementary Table 5 - xlsx file
- Supplementary Table 6 - xlsx file
- Supplementary Table 7 - xlsx file
