# Supplementary material for: Assessing Diversity of DNA Structure-Related Sequence Features in Prokaryotic Genomes
Source: DNA Res. 2014 Jan 9;21(3):285–97. doi: 10.1093/dnares/dst057 (PMC4060949; doi:10.1093/dnares/dst057)
Supplement: Supplementary Data [file supp_dst057_dst057supp.pdf]

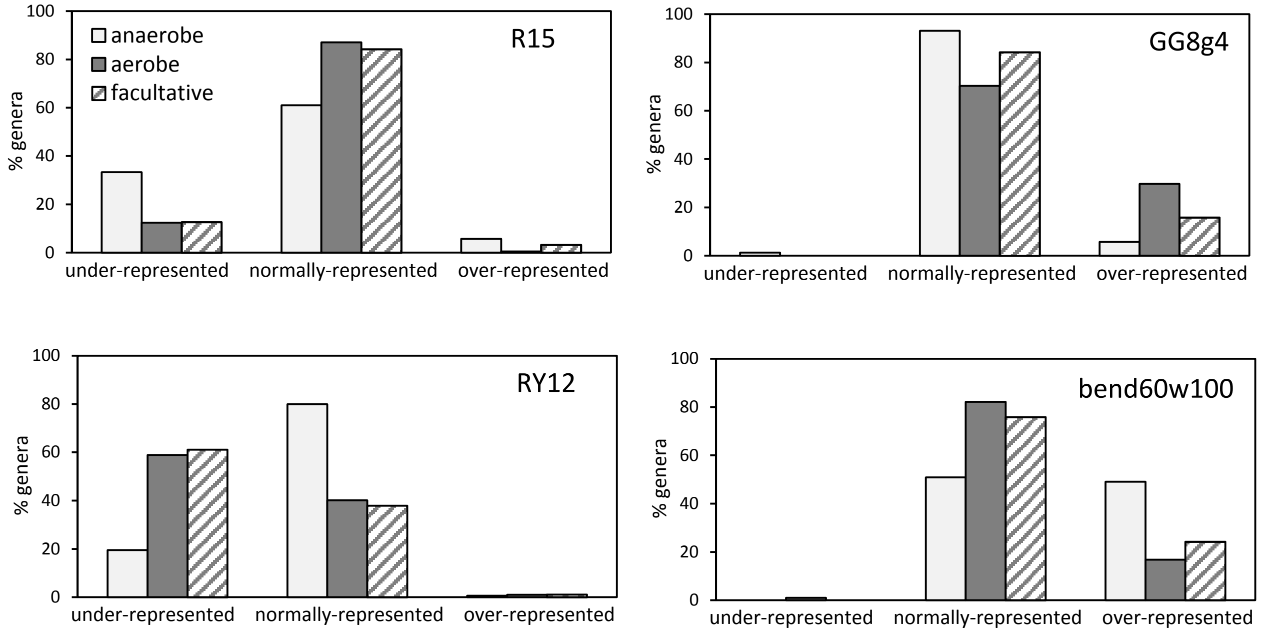

Figure S1. Comparison of pattern representations in different oxygen requirement classes for selected patterns. Bars show the percentage of species in each class which have the given pattern under-represented, normally-represented or over-represented. See legend to Figure 1.

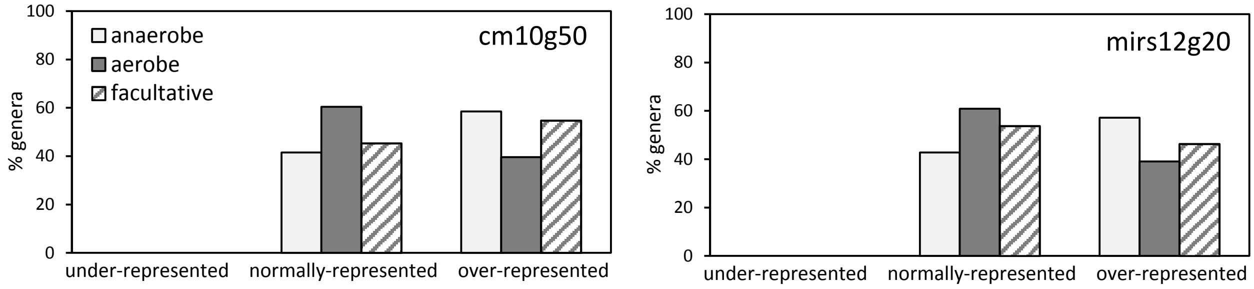

Figure S2. Comparison of representations of two forms of mirror repeats in the protein-coding regions for different oxygen requirement classes. Bars show the percentage of species in each class which have the given pattern under-represented, normally-represented or over-represented. See legend to Figure 1.

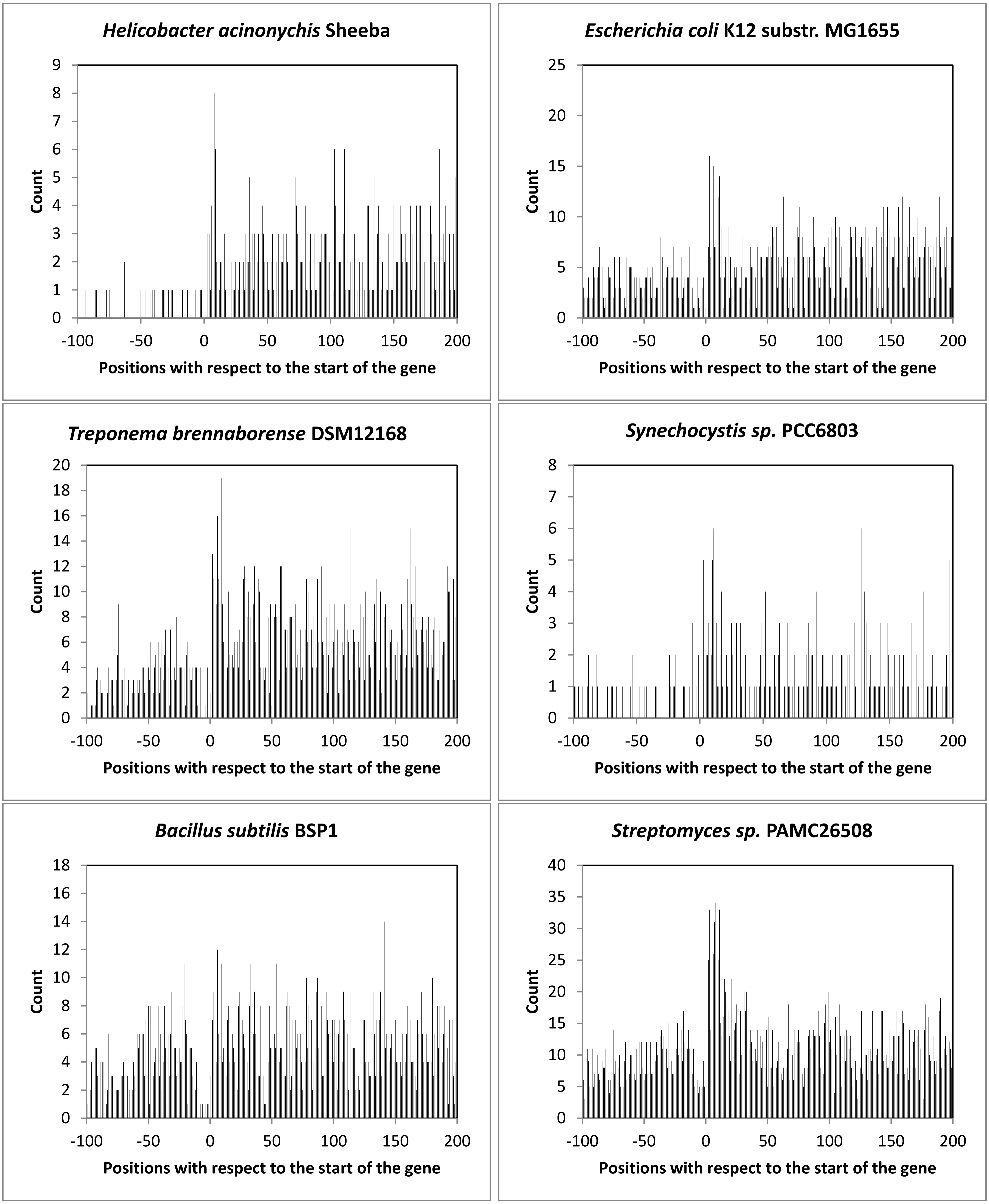

Figure S3 (a). Distribution of RY-patterns with respect to the start of the gene in selected genomes. Position zero refers to the first base of the start codon. The ordinate shows the counts of RY-patterns with the right end located at the position indicated by the abscissa.

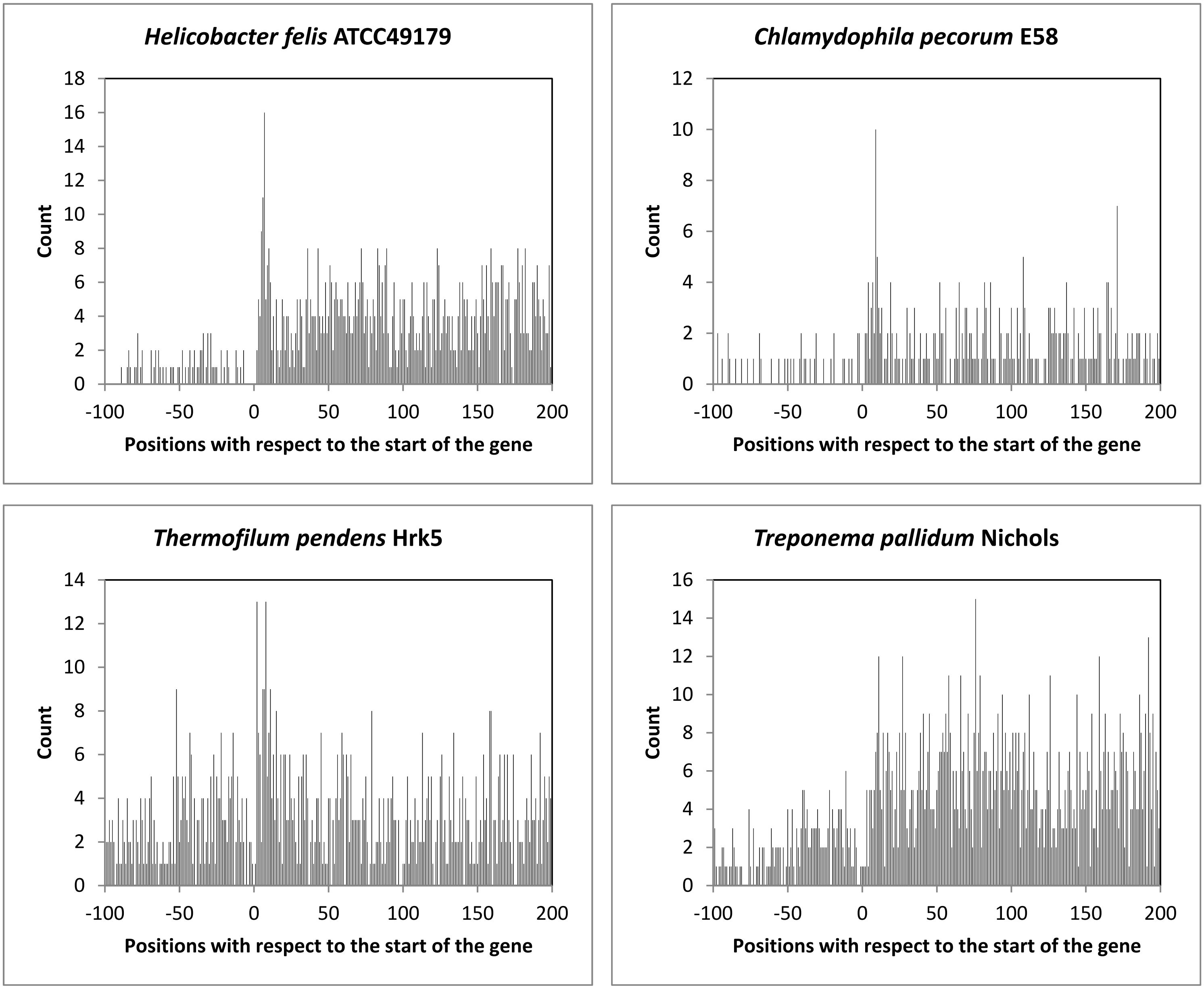

Figure S3 (b). Distribution of RY patterns with respect to the start of the gene in the genomes with the most overrepresented RY patterns.

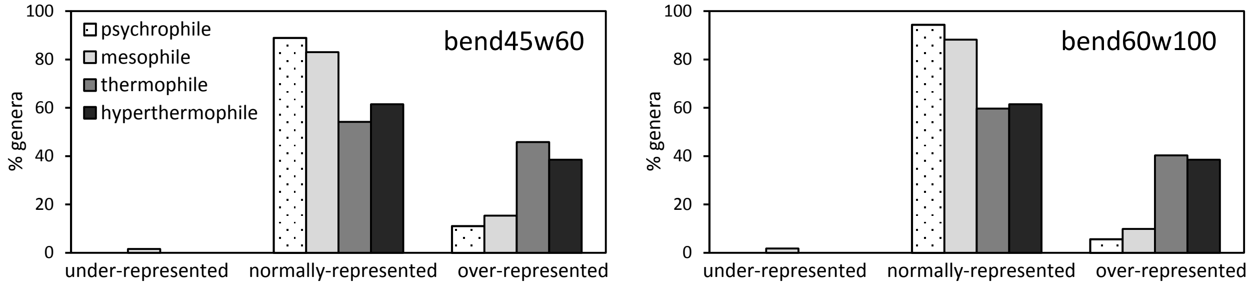

(a) Protein coding regions

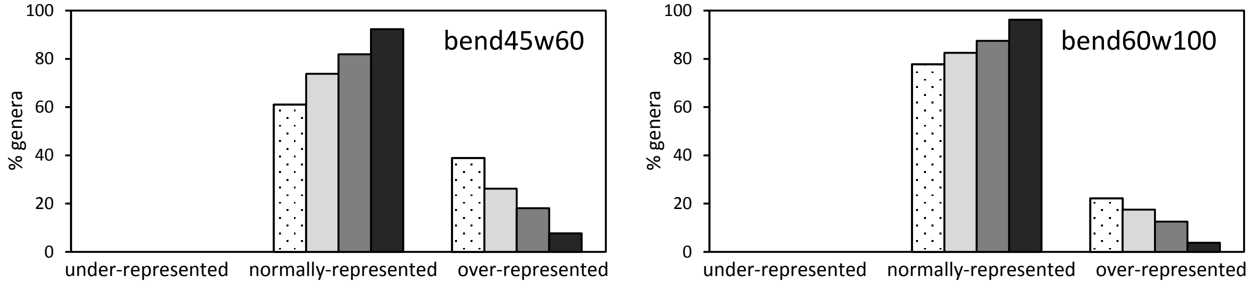

(b) Intergenic regions

Figure S4. Representations of intrinsic bends in different temperature classes (a) in protein coding regions and (b) in non-coding regions.

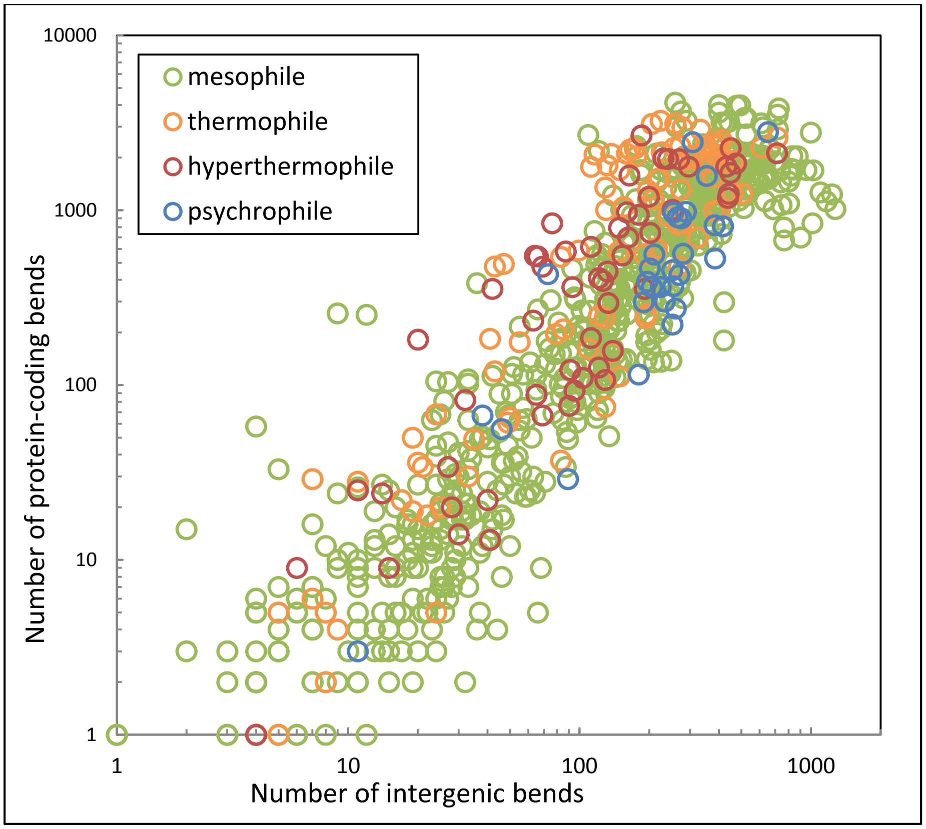

Figure S5. Comparison of number of the bend45w60 patterns in protein-coding regions and non-coding regions. Each circle represents one species and different colors refer to different OGT classes.
