# Supplementary material for: Assessing Diversity of DNA Structure-Related Sequence Features in Prokaryotic Genomes
Source: DNA Res. 2014 Jan 9;21(3):285–97. doi: 10.1093/dnares/dst057 (PMC4060949; doi:10.1093/dnares/dst057)
Supplement: Supplementary Data [file supp_dst057_dst057supp_table8.pdf]

Table S8. Pattern representation classes

General level	Detailed level	p-value	r = observed/expected
Under-represented	-4	$p \leq 10^{-12}$	$r \leq 0.67$
	-3	$p \leq 10^{-7}$	$r \leq 0.80$
	-2	$p \leq 10^{-4}$	$r \leq 0.91$
normally-represented	-1	$p \leq 10^{-2}$	$r \leq 0.95$
	0	$p > 10^{-2}$	$0.95 < r < 1.05$
	1	$p \leq 10^{-2}$	$r > 1.05$
Over-represented	2	$p \leq 10\text{-}^{4}$	$r >1.10$
	3	$p \leq 10^{-7}$	$r > 1.25$
	4	$p \leq 10^{-12}$	$r > 1.50$

The sequence patterns are classified into three general categories and nine detailed categories based on their representation in the genome using the p-value and the observed/expected ratio. A patern is assigned the most extreme category for which it qualifies by both criteria. See Materials and Methods for details and justification.

Table S9. Representation of sequence patterns in different phyla

Pattern	Pattern	AlPr	BePr	GaPr	DePr	EpPr	Firm	Acti	Cyan	Bact	Chlb	Chlf	Dein	Fuso	Chla	Spir	Acid	Verr	Defe	Plan	Aqui	Ther	Eury	Cren
name	code	58	39	76	23	11	61	63	12	34	5	8	6	5	6	4	4	4	4	4	8	5	44	16
Simple sequence repeats	1n8	-4.00	-3.00	-4.00	-4.00	-4.00	-4.00	-4.00	-4.00	-4.00	-167	-4.00	-4.00	-4.00	-3.79	-3.75	-3.00	-3.00	-4.00	-3.50	-4.00	-4.00	-4.00	-4.00
	2n5	-2.21	-2.00	-3.00	-3.00	-4.00	-4.00	-3.00	-3.63	-4.00	-3.00	-3.00	-2.60	-4.00	-4.00	-3.50	-2.00	-2.50	-4.00	-2.00	-4.00	-4.00	-3.00	-3.00
	3n4	-0.78	-2.00	-2.09	-100	-3.00	-3.00	-2.00	-150	-2.67	-2.00	-150	-150	-4.00	-3.00	-3.17	-0.25	-100	-3.50	-100	-3.50	-4.00	-3.00	-3.00
	4n4	0.00	0.00	0.00	0.00	0.00	0.00	0.00	0.00	0.00	0.00	0.00	0.00	-100	0.00	0.00	0.00	0.00	0.00	0.00	0.00	0.00	0.00	0.00
	5n4	0.00	0.00	0.00	0.00	0.00	0.00	0.00	0.00	0.00	0.00	0.00	0.00	0.00	0.00	0.07	0.00	0.00	0.00	0.00	0.00	0.00	0.00	0.00
	6n3	0.00	0.00	0.00	0.00	0.00	0.00	0.00	0.00	0.00	0.00	0.00	0.00	0.00	0.00	0.07	0.00	0.00	0.00	0.00	0.00	0.00	0.00	0.00
	7n3	0.00	0.00	0.00	0.00	0.00	0.00	0.00	0.00	0.75	0.00	100	0.00	0.00	0.00	0.00	0.00	0.50	100	2.00	0.00	0.00	0.00	0.00
	8n3	0.00	0.00	0.00	0.00	0.00	0.00	0.00	0.00	0.00	0.00	0.00	0.00	0.00	0.00	0.50	0.00	0.00	0.50	0.00	0.00	0.00	0.00	0.00
	9n2	0.00	0.00	0.00	0.00	0.00	0.00	0.00	0.00	0.00	0.00	0.00	0.00	0.00	0.00	-0.36	0.00	0.00	0.00	0.00	0.00	0.00	0.00	0.00
	10n2	0.00	0.00	0.00	0.00	0.00	0.00	0.00	0.00	0.00	0.00	0.00	0.00	0.00	0.00	0.17	0.00	0.00	0.00	0.00	0.00	100	0.00	0.00
	11n2	0.00	0.00	0.00	0.00	0.00	0.00	0.00	0.59	0.00	0.00	0.00	0.00	0.00	0.00	0.00	0.00	0.00	0.00	0.00	0.00	0.00	0.00	0.00
Close direct repeats	4n6g12	0.00	0.00	0.00	0.00	0.00	0.00	100	0.00	0.00	0.00	0.00	0.00	0.00	0.00	0.13	0.00	0.00	0.00	125	0.00	0.00	0.00	0.00
	6n6g24	0.42	0.25	0.88	100	0.00	0.30	2.00	150	100	100	100	0.00	0.00	0.00	0.98	150	0.00	150	125	0.00	0.00	0.10	0.00
	8n4g24	0.50	100	114	2.00	0.00	100	3.00	3.00	150	2.00	100	0.00	0.00	0.00	125	2.25	0.50	150	2.75	0.00	0.00	100	0.00
	cd8g6	0.00	0.00	0.00	100	100	0.00	2.00	192	0.00	0.00	0.75	120	0.00	0.00	0.02	0.00	0.00	0.00	0.00	0.00	100	0.60	100
	cd10g50	0.00	0.00	0.00	0.00	0.00	0.00	100	0.60	0.00	0.00	0.00	0.00	0.00	-0.19	0.15	0.00	0.00	-100	100	0.00	0.00	0.00	0.00
Palindromes & inverted repeats	cp8g6	3.00	3.00	4.00	3.50	3.00	4.00	3.00	181	3.00	2.00	4.00	2.63	3.00	4.00	2.86	3.50	4.00	3.00	150	2.00	4.00	2.00	2.00
	cp10g50	3.00	4.00	4.00	4.00	3.83	4.00	4.00	3.54	4.00	4.00	4.00	3.75	4.00	4.00	3.69	3.50	2.00	3.00	3.50	100	4.00	100	100
	pals9	109	2.00	3.35	3.00	2.00	4.00	4.00	2.00	4.00	3.00	3.50	3.00	3.00	3.96	2.17	150	2.00	150	100	0.00	3.50	0.10	0.00
	pals9g12	3.66	4.00	4.00	4.00	3.72	4.00	4.00	3.71	4.00	4.00	4.00	3.75	4.00	4.00	3.77	4.00	4.00	3.00	4.00	100	4.00	2.00	100
	pals12g20	4.00	4.00	4.00	4.00	4.00	4.00	4.00	4.00	4.00	4.00	4.00	4.00	4.00	4.00	3.77	4.00	4.00	3.00	4.00	0.00	4.00	100	0.22
H-DNA-related patterns	cm8g6	0.00	0.00	0.00	0.00	0.00	0.00	0.00	0.00	0.00	0.00	0.00	0.00	0.00	0.00	0.34	0.00	0.00	0.00	0.00	0.00	0.00	0.00	0.00
	cm10g50	0.00	0.00	0.00	0.00	0.00	0.00	0.00	0.00	0.00	0.00	0.00	0.00	0.00	0.00	0.34	0.25	0.00	0.00	0.00	0.00	0.00	0.00	0.00
	mirs9	0.00	0.00	0.00	0.00	0.00	0.00	0.00	0.00	0.00	0.00	0.00	0.00	0.00	0.00	0.00	0.00	0.00	0.00	0.00	0.00	0.00	0.00	0.00
	mirs9g12	0.00	0.00	0.00	0.00	0.00	0.00	0.25	0.17	0.00	0.00	0.34	0.00	0.00	0.00	0.25	0.50	0.00	0.00	0.25	0.00	0.00	0.00	0.00
	mirs12g20	0.00	0.00	0.00	0.00	0.00	0.00	0.00	0.09	0.00	0.00	0.00	0.00	0.00	0.00	0.13	0.00	0.50	0.00	0.25	0.00	0.00	0.00	0.00
	R15	-0.20	0.00	0.00	0.00	-100	-0.20	0.00	-107	0.00	0.00	0.25	-100	-4.00	0.00	-0.90	-0.50	100	-3.00	0.00	-2.00	-3.00	-100	-100
	R30	0.00	0.00	0.00	0.00	0.00	0.00	0.00	0.00	0.00	0.00	0.00	0.00	0.00	0.00	0.38	0.00	0.00	0.00	0.00	0.00	0.00	0.00	0.00
	R30e3	0.00	0.00	0.00	0.00	0.00	0.00	0.00	0.00	0.00	0.00	0.00	0.00	-2.00	-0.25	-0.13	0.00	0.50	-100	0.00	-0.50	-150	0.00	0.00
	R45e6	0.00	0.00	0.00	0.00	0.00	0.00	0.00	0.00	0.00	0.00	0.00	0.00	-2.00	0.00	0.15	0.00	0.50	0.00	0.50	-0.50	0.00	0.00	0.00
	R60e9	0.00	0.00	0.00	0.00	0.00	0.00	0.00	0.00	0.00	0.00	0.00	0.00	0.00	0.00	0.07	0.00	0.00	0.00	0.50	0.00	0.00	0.00	0.00
G-DNA-related patterns	GG8g4	100	133	0.00	0.00	0.00	0.00	100	100	0.00	0.00	0.00	2.70	0.00	0.00	0.54	100	0.00	0.00	150	0.00	0.00	0.00	0.00
	GGG4g6	0.00	0.00	0.00	0.00	0.00	-0.63	0.00	0.00	0.00	0.00	-0.31	100	0.00	0.00	-0.25	0.00	0.00	0.00	0.00	-0.50	0.00	-0.42	0.00
	GGGG4g6	0.00	0.00	0.00	0.00	0.00	0.00	0.00	0.00	0.00	0.00	0.17	0.00	0.00	0.00	0.00	0.00	0.00	0.00	0.00	0.00	0.00	0.00	0.00
Z-DNA-related patterns	GC6	-100	-100	-2.00	-2.00	-100	-100	-2.00	-2.00	-100	-4.00	-2.00	-120	0.00	0.00	-0.77	-150	-100	-0.50	-2.75	-0.25	0.00	-2.00	-100
	GC8	-0.50	-100	-100	-100	0.00	0.00	-3.00	-0.25	0.00	-2.00	-134	-125	0.00	0.00	0.00	-0.50	0.00	0.00	-3.00	0.00	0.00	0.00	0.00
	RY12	-2.50	-2.00	-3.00	-100	-0.89	-0.33	-2.00	-2.00	-0.35	-2.00	-3.25	-0.25	0.00	0.42	-0.09	-0.75	0.00	-100	-3.00	0.00	0.00	-0.72	0.00
	RY12e1	-2.00	-140	-3.00	-100	-100	-0.25	-2.00	-3.00	0.00	-3.00	-2.25	0.00	0.00	0.84	0.05	-0.75	0.00	-100	-3.00	0.50	0.00	-0.64	-0.98
	RY18e2	-2.65	-2.00	-2.53	-100	0.00	0.00	-2.00	-2.00	0.00	-2.00	-3.00	-100	0.00	0.75	0.00	-0.75	0.00	0.00	-3.50	0.00	0.00	0.00	0.00
	RY24e3	-103	-150	-100	0.00	0.00	0.00	-100	-100	0.00	-100	-125	0.00	0.00	0.00	-0.17	-100	0.00	0.00	-100	0.00	0.00	0.00	0.00
DNA Bending	bend45w60	0.28	0.00	0.07	100	3.00	2.00	0.00	150	2.00	100	0.17	0.45	2.00	150	0.50	0.00	0.00	2.50	0.00	2.00	2.00	2.00	0.00
	bend60w100	0.00	0.00	0.00	0.50	2.97	2.00	0.00	100	2.00	0.00	0.00	0.00	2.00	0.75	0.25	0.00	0.00	3.00	0.00	2.25	3.00	2.00	0.00
	bend90w120	0.00	0.00	0.00	0.00	3.00	100	0.00	100	100	0.00	0.00	0.00	3.00	0.00	0.20	0.00	0.00	3.50	0.00	2.00	3.00	100	0.00

Numbers in the table refer to the medians of pattern representation among all genera of the corresponding phylum. The pattern representations were categorized into 9 categories from -4 (extremely under-represented), through 0 (normally represented), to +4 (extremely over-represented). See Materials and Methods for details. Codes in the second column refer to specific sequence patterns (see **Error! Reference source not found.**). Columns represent different phyla abbreviated as follows: AlPr, α-proteobacteria; BePr, β-Proteobacteria; GaPr, γ-Proteobacteria; DePr, δ-Proteobacteria; EpPr, ε-Proteobacteria; Firm, Firmicutes; Acti, Actinobacteria; Cyan, Cyanobacteria; Bact, Bacteroidetes; Chlb, Chlorobi; Chlf, Chloroflexi; Dein, Deinococcus-Thermus; Fuso, Fusobacteria; Chla, Chlamydiae; Spir, Spirochaetes; Acid, Acidobacteria; Verr, Verrucomicrobia; Defe, Deferribacteres; Plan, Planctomycetes; Aqui, Aquificales; Ther, Thermotogae; Eury, Euryarchaeota; Cren, Crenarchaeota. Numbers in the second row indicate the number of genera available for each phylum. Only phyla represented by three or more genera are shown.

Table S10. Representation of sequence patterns in the protein-coding regions for different phyla

Pattern name	Pattern code	AlPr	BePr	GaPr	DePr	EpPr	Firm	Acti	Cyan	Bact	Chlb	Chlf	Dein	Fuso	Chla	Spir	Acid	Verr	Defe	Plan	Aqui	Ther	Eury	Cren
		58	39	76	23	11	61	63	12	34	5	8	6	5	6	4	4	4	4	4	8	5	44	16
Simple sequence repeats	1n8	-3.00	-3.00	-4.00	-3.20	-4.00	-4.00	-3.50	-4.00	-4.00	-2.58	-4.00	-4.00	-4.00	-4.00	-3.75	-3.00	-3.00	-4.00	-3.00	-4.00	-4.00	-4.00	-4.00
	2n5	-2.00	-100	-2.96	-2.50	-4.00	-3.45	-3.00	-3.00	-4.00	-3.00	-2.50	-2.00	-4.00	-4.00	-3.43	-100	-2.50	-4.00	-175	-4.00	-4.00	-3.00	-3.00
	3n4	-0.97	-2.00	-2.00	-100	-3.00	-2.88	-2.00	-109	-2.00	-2.00	-144	-150	-4.00	-2.00	-2.59	-100	-150	-3.00	-0.50	-3.00	-3.00	-3.00	-3.00
	4n4	0.00	0.00	0.00	0.00	0.00	0.00	0.00	0.00	0.00	0.00	0.00	0.00	0.00	0.00	0.00	0.00	0.00	0.00	0.00	0.00	0.00	0.00	0.00
	5n4	0.00	0.00	0.00	0.00	0.00	0.00	0.00	0.00	0.00	0.00	0.00	0.00	0.00	0.00	0.00	0.00	0.00	0.00	0.00	0.00	0.00	0.00	0.00
	6n3	0.00	0.00	0.00	0.00	0.00	0.00	0.00	0.00	0.00	0.00	0.00	0.00	-100	0.00	0.00	0.00	0.00	0.00	0.00	0.00	0.00	0.00	0.00
	7n3	0.00	0.00	0.00	0.00	0.00	0.00	0.00	0.00	0.00	0.00	0.00	0.00	0.00	0.00	0.00	0.00	0.00	0.00	0.00	0.00	0.00	0.00	0.00
	8n3	0.00	0.00	0.00	0.00	0.00	0.00	0.00	0.00	0.00	0.00	0.00	0.00	0.00	0.00	0.00	0.00	0.00	0.00	0.00	0.00	0.00	0.00	0.00
	9n2	0.00	0.00	0.00	0.00	0.00	0.00	0.00	0.00	0.00	0.00	0.00	0.00	0.00	0.00	-0.22	0.00	0.00	0.00	0.00	0.00	-0.50	0.00	0.00
	10n2	0.00	0.00	0.00	0.00	0.00	0.00	0.00	0.00	0.00	0.00	0.00	0.00	0.00	0.00	0.00	0.00	0.00	0.00	0.00	0.00	0.00	0.00	0.00
	11n2	0.00	0.00	0.00	0.00	0.00	0.00	0.00	0.00	0.00	0.00	0.00	0.00	0.00	0.00	0.00	0.00	0.00	0.00	0.00	0.00	0.00	0.00	0.00
Close direct repeats	4n6g12	0.00	0.00	0.00	0.00	0.00	0.00	0.20	0.00	0.00	0.00	0.50	0.00	0.00	0.00	0.13	0.00	0.00	0.00	0.50	0.00	0.00	0.00	0.00
	6n6g24	0.00	0.00	0.00	0.00	0.00	0.00	2.00	175	0.00	0.92	0.50	0.00	0.00	0.00	0.47	0.00	0.00	0.00	0.50	0.00	0.00	0.30	0.00
	8n4g24	0.04	0.33	0.39	0.50	0.00	0.00	2.00	2.00	0.00	0.00	0.00	0.00	0.00	0.00	0.47	0.00	0.00	0.00	150	0.00	0.00	0.42	0.00
	cd8g6	0.00	0.00	0.00	0.00	0.50	0.00	100	0.00	0.00	0.00	0.00	100	0.00	0.00	-0.25	0.00	0.00	0.00	0.00	0.00	0.00	0.00	0.13
	cd10g50	-0.33	-0.17	-100	0.00	0.00	-100	0.00	0.00	0.00	0.00	0.00	0.00	0.00	-0.81	-0.47	0.00	-0.50	-100	0.00	-100	-129	-0.50	0.00
Palindromes & inverted repeats	cp8g6	0.00	0.00	0.00	0.00	0.00	0.00	0.00	0.00	0.00	0.00	0.00	0.00	0.00	150	0.15	-0.50	0.00	0.00	0.00	0.00	0.00	0.00	0.00
	cp10g50	-0.25	0.00	0.00	0.00	0.50	0.00	0.00	0.00	0.00	0.00	0.00	0.00	0.00	0.50	0.17	0.00	-0.50	0.00	0.25	0.00	100	0.00	0.00
	pals9	0.00	0.00	0.00	0.00	0.00	0.00	0.00	0.00	0.00	0.00	0.00	0.00	0.00	0.44	0.21	0.00	0.00	0.00	0.00	0.00	0.00	0.00	0.00
	pals9g12	0.00	0.00	0.03	0.00	0.67	0.00	0.00	0.00	0.00	0.00	0.75	0.00	0.00	150	0.92	0.00	0.00	0.00	0.50	0.00	0.00	0.00	0.00
	pals12g20	0.00	0.00	0.00	0.00	0.00	0.00	0.00	0.00	0.00	0.00	0.00	0.00	0.00	0.00	0.15	0.00	-0.50	0.00	0.50	0.00	0.00	0.00	0.00
H-DNA-related patterns	cm8g6	0.00	0.00	0.00	0.00	0.00	0.00	0.00	0.00	0.00	0.00	0.00	0.00	100	0.00	0.25	0.00	0.50	0.00	0.00	0.00	0.00	0.00	0.00
	cm10g50	100	2.00	2.00	2.00	150	2.00	0.00	122	3.00	4.00	2.17	0.00	2.00	2.00	2.15	0.75	2.00	100	125	2.00	0.14	171	0.18
	mirs9	0.00	0.00	0.00	0.00	0.00	0.00	0.00	0.00	0.00	0.00	0.00	0.00	0.00	0.00	0.00	0.00	0.00	0.00	0.00	0.00	0.00	0.00	0.00
	mirs9g12	0.00	0.00	0.33	100	0.00	0.39	0.00	0.00	0.00	142	0.71	0.00	100	0.04	0.42	0.00	100	0.00	0.25	0.25	0.00	0.00	0.00
	mirs12g20	100	2.00	167	2.00	100	2.00	0.00	116	2.25	3.00	175	0.00	2.00	184	179	0.50	150	2.00	0.25	125	100	150	0.00
	R15	0.00	0.00	0.00	0.00	-100	-0.60	0.00	-0.75	0.00	0.00	0.00	-100	-4.00	0.00	-0.72	-0.50	0.00	-2.50	0.00	-2.25	-3.00	-100	-100
	R30	0.00	0.00	0.00	0.00	0.00	0.00	0.00	0.00	0.00	0.00	0.00	0.00	0.00	0.00	0.25	0.00	0.50	0.00	0.00	0.00	0.00	0.00	0.00
	R30e3	0.00	0.00	0.00	0.00	0.00	0.00	0.00	0.00	0.00	0.00	0.00	0.00	-3.00	0.00	-0.37	0.00	0.50	-100	0.00	-0.50	-2.00	0.00	0.00
	R45e6	0.00	0.00	0.00	0.00	0.00	0.00	0.00	0.00	0.00	0.00	0.00	0.00	-2.00	0.00	0.15	0.00	100	-0.50	0.00	0.00	-0.50	0.00	0.00
	R60e9	0.00	0.00	0.00	0.00	0.00	0.00	0.00	0.00	0.00	0.00	0.00	0.00	-100	0.00	0.04	0.00	100	0.00	0.00	0.00	-0.50	0.00	0.00
G-DNA-related patterns	GG8g4	100	133	0.00	0.94	0.00	0.00	100	0.75	0.00	0.00	0.50	2.60	0.00	0.00	0.34	0.50	0.00	0.00	2.00	0.00	0.00	0.00	0.00
	GGG4g6	0.00	0.00	0.00	0.00	0.00	0.00	0.00	0.00	0.00	0.00	0.00	140	0.00	0.00	0.00	0.00	0.00	0.00	0.00	0.00	0.00	0.00	0.00
	GGGG4g6	0.00	0.00	0.00	0.00	0.00	0.00	0.00	0.00	0.00	0.00	0.00	0.00	0.00	0.00	0.00	0.00	0.00	0.00	0.00	0.00	0.00	0.00	0.00
Z-DNA-related patterns	GC6	0.00	0.00	-159	-0.57	-0.50	-0.58	-2.00	-2.00	0.00	-3.25	-188	-120	0.00	0.00	-0.22	-0.75	-100	-0.50	-150	0.00	0.00	-100	-0.50
	GC8	0.00	0.00	-0.54	0.00	0.00	0.00	-3.00	0.00	0.00	-142	-0.67	-0.88	0.00	0.00	0.00	-0.50	0.00	0.00	-2.00	0.00	0.00	0.00	0.00
	RY12	-2.00	-193	-2.00	-0.50	-0.67	0.00	-100	-100	0.00	-2.00	-2.00	0.00	0.00	0.09	0.00	0.00	0.00	0.00	-2.00	0.00	0.00	-0.66	0.00
	RY12e1	-2.00	-133	-2.71	-100	-0.42	-100	-100	-3.00	0.00	-3.00	-2.00	0.00	-100	134	0.00	-0.75	0.00	-0.50	-3.00	0.50	0.00	-0.45	-100
	RY18e2	-2.00	-150	-2.00	0.00	0.00	0.00	-100	-0.50	0.00	-2.00	-2.59	-0.25	0.00	100	0.00	-125	0.00	0.00	-2.50	0.00	0.00	0.00	-0.54
	RY24e3	-100	-100	-100	0.00	0.00	0.00	-0.44	0.00	0.00	-100	-100	-0.25	0.00	0.00	0.00	-0.50	-0.50	0.00	-0.50	0.00	0.00	0.00	0.00
DNA Bending	bend45w60	0.00	0.00	0.00	0.00	2.50	100	0.00	100	100	0.00	0.00	0.00	2.00	0.00	-0.02	-100	0.00	2.00	-0.75	2.00	2.00	100	0.00
	bend60w100	0.00	0.00	0.00	0.00	2.00	0.00	0.00	0.00	0.00	0.00	0.00	0.00	2.00	0.00	-0.17	0.00	0.00	2.50	-0.50	2.00	2.00	0.65	0.00
	bend90w120	0.00	0.00	0.00	0.00	120	0.00	0.00	0.00	0.00	0.00	0.00	0.00	2.00	0.00	-0.79	0.00	0.00	3.00	0.00	0.75	2.00	0.00	0.00

Same as Table S9 but showing the data for protein-coding regions only. Protein-coding regions are defined as all segments annotated as protein coding sequences (the “CDS” feature in the GenBank entry), whereas all other segments are considered non-coding. That is, untranslated regions of genes as well as RNA genes are considered “intergenic” for the purpose of this analysis.

Table S11. Representation of sequence patterns in the intergenic regions for different phyla

Pattern name	Pattern code	AlPr	BePr	GaPr	DePr	EpPr	Firm	Acti	Cyan	Bact	Chlb	Chlf	Dein	Fuso	Chla	Spir	Acid	Verr	Defe	Plan	Aqui	Ther	Eury	Cren
		58	39	76	23	11	61	63	12	34	5	8	6	5	6	4	4	4	4	4	8	5	44	16
Simple sequence repeats	1n8	-3.00	-2.00	-2.00	-1.00	-2.00	-3.00	-3.00	-3.00	-2.00	1.00	-2.50	-3.00	-2.00	-0.34	-1.79	-1.50	-2.00	-1.50	-2.00	-3.00	-4.00	-3.00	-3.75
	2n5	-1.00	-1.75	-2.00	-1.25	-3.00	-2.50	-2.00	-2.00	-3.00	-2.00	-1.69	-2.00	-3.00	-2.79	-2.29	-0.75	-2.00	-3.00	-1.00	-2.00	-3.00	-2.00	-2.00
	3n4	0.00	0.00	0.00	0.00	-1.00	-0.50	0.00	-0.17	-1.00	0.00	0.00	0.00	-1.00	-0.59	-0.72	0.00	0.00	-1.00	0.00	0.00	-1.00	0.00	0.00
	4n4	0.00	0.00	0.00	0.00	0.00	0.00	0.00	0.00	0.00	0.00	0.00	0.00	0.00	0.00	0.00	0.00	0.00	0.00	0.00	0.00	0.00	0.00	0.00
	5n4	0.00	0.00	0.00	0.00	0.00	0.00	0.00	0.00	0.00	0.00	0.00	0.00	0.00	0.00	0.00	0.00	0.00	0.00	0.00	0.00	0.00	0.00	0.00
	6n3	0.00	0.00	0.00	0.00	0.00	0.00	0.00	0.00	0.00	0.00	0.00	0.00	0.00	0.00	0.04	0.00	0.00	0.00	0.00	0.00	0.00	0.00	0.00
	7n3	0.00	0.00	0.00	0.00	0.00	0.00	0.00	0.00	0.00	0.00	1.17	0.00	0.00	0.00	0.00	0.00	1.50	2.00	0.25	0.00	0.00	0.00	0.00
	8n3	0.00	0.00	0.00	0.00	0.00	0.00	0.00	0.00	0.00	0.00	0.00	0.00	0.00	0.00	0.34	0.00	0.00	0.50	0.00	0.00	0.00	0.00	0.00
	9n2	0.00	0.00	0.00	0.00	0.00	0.00	0.00	0.00	0.00	0.00	0.00	0.00	0.00	0.00	0.00	0.00	0.00	0.00	0.00	0.00	0.00	0.00	0.00
	10n2	0.00	0.00	0.00	0.00	0.00	0.00	0.00	0.09	0.00	0.00	0.00	0.00	0.00	0.00	0.00	0.00	0.00	0.00	0.00	0.00	0.00	0.00	0.00
	11n2	0.00	0.00	0.00	0.00	0.00	0.00	0.00	0.41	0.00	0.00	0.00	0.00	0.00	0.00	0.16	0.75	0.00	0.00	0.00	0.00	1.00	0.00	0.00
Close direct repeats	4n6g12	0.00	0.00	0.00	0.00	0.00	0.00	0.20	0.03	0.00	0.00	2.00	0.00	0.00	0.00	0.31	0.00	0.00	1.00	0.00	0.00	0.00	0.00	0.00
	6n6g24	0.00	0.00	0.16	0.47	0.00	0.00	0.00	0.00	0.75	0.00	0.50	0.00	0.00	0.00	0.68	0.50	0.00	2.00	0.00	0.00	0.00	0.00	0.00
	8n4g24	0.00	0.00	0.50	1.00	0.00	0.00	2.00	0.54	1.25	1.42	2.00	0.00	0.00	0.00	0.88	1.00	0.00	2.00	0.50	0.00	0.00	0.00	0.00
	cd8g6	0.47	1.00	0.56	1.00	1.00	1.00	2.00	3.50	1.00	1.00	2.84	1.23	0.00	0.00	0.69	2.00	1.50	2.00	0.25	0.00	1.50	1.00	1.00
	cd10g50	1.27	1.00	1.30	2.00	1.00	1.25	3.73	2.50	3.00	1.00	4.00	1.55	0.00	0.00	1.07	3.50	1.50	0.50	1.50	0.00	2.00	2.00	1.00
Palindromes & inverted repeats	cp8g6	4.00	4.00	4.00	4.00	4.00	4.00	4.00	3.21	4.00	4.00	4.00	4.00	4.00	4.00	3.63	4.00	4.00	4.00	4.00	3.50	4.00	3.75	2.00
	cp10g50	4.00	4.00	4.00	4.00	3.50	4.00	4.00	4.00	4.00	4.00	4.00	4.00	4.00	4.00	4.00	4.00	4.00	4.00	4.00	2.50	4.00	3.00	1.88
	pals9	1.00	1.00	1.54	1.00	0.00	2.00	2.00	0.84	2.00	1.00	1.00	1.00	1.00	1.00	0.62	0.25	1.00	0.50	0.00	0.00	1.50	0.00	0.00
	pals9g12	4.00	3.75	4.00	4.00	2.00	4.00	4.00	3.50	4.00	4.00	4.00	3.90	4.00	4.00	3.42	4.00	3.50	2.50	4.00	1.00	4.00	1.75	0.50
	pals12g20	1.88	1.00	2.00	2.00	0.00	3.00	4.00	1.00	3.00	2.00	2.00	1.50	2.00	1.63	1.07	1.50	1.50	0.00	1.50	0.00	1.50	0.00	0.00
H-DNA-related patterns	cm8g6	0.00	0.00	0.00	0.00	0.00	0.00	0.00	0.17	0.00	0.00	0.00	0.00	0.00	0.00	0.00	0.00	0.00	0.00	0.00	0.00	0.00	0.00	0.00
	cm10g50	0.00	0.00	0.00	0.00	0.00	0.00	0.00	0.00	0.00	0.00	0.00	0.00	0.00	0.00	0.00	0.00	0.00	0.00	0.00	0.00	0.00	0.00	0.00
	mirs9	0.00	0.00	0.00	0.00	0.00	0.00	0.00	0.00	0.00	0.00	0.00	0.00	0.00	0.00	0.00	0.00	0.00	0.00	0.00	0.00	0.00	0.00	0.00
	mirs9g12	0.00	0.00	0.00	0.00	0.00	0.00	0.00	0.00	0.00	0.00	0.00	0.00	0.00	0.00	0.00	0.00	0.00	0.00	0.00	0.00	0.00	0.00	0.00
	mirs12g20	0.00	0.00	0.00	0.00	0.00	0.00	0.00	0.00	0.00	0.00	0.00	0.00	0.00	0.00	0.00	0.00	0.00	0.00	0.00	0.00	0.00	0.00	0.00
	R15	0.00	0.00	0.00	0.00	0.00	0.00	0.00	-0.42	0.00	0.00	0.00	0.00	0.00	0.00	0.09	0.00	0.00	0.00	0.00	0.00	0.00	0.00	0.00
	R30	0.00	0.00	0.00	0.00	0.00	0.00	0.00	0.00	0.00	0.00	0.00	0.00	0.00	0.00	0.00	0.00	0.00	0.00	0.00	0.00	0.00	0.00	0.00
	R30e3	0.00	0.00	0.00	0.00	0.00	0.00	0.00	0.00	0.00	0.00	0.00	0.00	0.00	0.00	0.11	0.00	0.00	0.00	0.00	0.00	0.00	0.00	0.00
	R45e6	0.00	0.00	0.00	0.00	0.00	0.00	0.00	0.00	0.00	0.00	0.00	0.00	0.00	0.00	0.18	0.00	0.00	0.00	0.00	0.00	0.00	0.00	0.00
	R60e9	0.00	0.00	0.00	0.00	0.00	0.00	0.00	0.00	0.00	0.00	0.00	0.00	0.00	0.00	0.16	0.00	0.00	0.00	0.00	0.00	0.00	0.00	0.00
G-DNA-related patterns	GG8g4	0.00	0.00	0.00	0.00	0.00	0.00	0.00	0.00	0.00	0.00	0.00	0.00	0.00	0.00	0.00	0.00	0.00	0.00	0.00	0.00	0.00	0.00	0.00
	GGG4g6	0.00	0.00	0.00	0.00	0.00	0.00	-0.52	0.00	0.00	0.00	0.25	-0.10	0.00	0.00	-0.07	0.00	-1.00	0.00	0.00	0.00	0.00	0.00	0.00
	GGGG4g6	0.00	0.00	0.00	0.00	0.00	0.00	0.00	0.00	0.00	0.00	0.00	0.00	0.00	0.00	0.00	0.00	0.00	0.00	0.00	0.00	0.00	0.00	0.00
Z-DNA-related patterns	GC6	-0.50	-1.06	-1.00	-1.00	0.00	-0.25	-2.00	-0.25	-1.00	-1.00	-1.23	0.00	0.00	0.00	-0.50	-1.25	-1.50	0.00	-2.50	0.00	0.00	-1.00	0.00
	GC8	0.00	-0.97	0.00	0.00	0.00	0.00	-1.25	0.00	0.00	0.00	0.00	0.00	0.00	0.00	0.00	-0.25	0.00	0.00	-1.00	0.00	0.00	0.00	0.00
	RY12	-1.00	-1.00	-1.00	0.00	0.00	0.00	-1.00	-0.34	0.00	-0.58	-1.50	0.00	0.00	0.00	0.11	-0.25	0.00	0.00	-1.00	0.00	0.00	0.00	0.00
	RY12e1	-1.88	-1.50	-2.45	-0.27	0.00	0.00	-2.00	-3.00	0.00	-0.58	-2.00	0.00	0.00	0.00	0.52	0.00	-0.50	-0.50	-3.00	0.00	0.00	0.00	0.00
	RY18e2	-0.75	-1.00	-1.00	0.00	0.00	0.00	-1.00	-0.34	0.00	-1.00	-1.25	0.00	0.00	0.00	0.17	-0.25	0.00	0.00	-1.50	0.00	0.00	0.00	0.00
	RY24e3	0.00	0.00	0.00	0.00	0.00	0.00	0.00	0.00	0.00	0.00	0.00	0.00	0.00	0.00	0.00	0.00	0.00	0.00	0.00	0.00	0.00	0.00	0.00
DNA Bending	bend45w60	0.00	0.00	0.94	0.00	2.50	1.70	0.00	1.42	1.00	0.25	0.00	0.00	1.00	0.71	1.00	0.00	0.00	1.00	0.75	0.00	1.00	1.00	0.00
	bend60w100	0.00	0.00	0.82	0.00	2.00	1.00	0.00	1.09	1.00	0.00	0.00	0.00	1.00	0.00	0.93	0.25	0.00	1.00	0.50	0.00	0.50	1.00	0.00
	bend90w120	0.00	0.00	0.08	0.00	1.50	1.00	0.00	0.09	1.00	0.00	0.00	0.00	1.00	0.00	0.69	0.00	0.00	1.00	0.00	0.00	0.50	0.00	0.00

Same as Table S9 but showing the data for ‘intergenic’ regions only, that is, all regions not annotated as ‘CDS’ in the GenBank files.

Table S12. Representation of sequence patterns in different OGT and oxygen requirement classes

Pattern name	Pattern code	Psychrophile	Mesophile	Thermophile	Hyperthermophile	Anaerobe	Aerobe	Facultative	Microaerophile
		18	382	72	26	159	202	95	9
Simple sequence repeats	1n8	-3.83	-3.54	-3.89	-4.00	-3.73	-3.61	-3.65	-3.67
	2n5	-3.15	-2.89	-3.40	-3.45	-3.30	-2.74	-3.02	-3.11
	3n4	-1.93	-1.92	-2.83	-3.09	-2.56	-1.75	-2.15	-2.33
	4n4	-0.06	0.01	-0.06	-0.08	-0.04	0.01	0.00	-0.10
	5n4	0.00	0.07	0.00	0.00	0.02	0.10	0.01	0.11
	6n3	0.20	0.19	0.06	-0.03	0.05	0.25	0.08	0.31
	7n3	1.27	0.48	0.45	0.00	0.42	0.59	0.35	0.89
	8n3	0.56	0.30	0.12	0.01	0.25	0.27	0.20	0.33
	9n2	-0.08	0.09	0.22	0.04	0.09	0.19	-0.11	-0.11
	10n2	0.09	0.25	0.25	0.28	0.27	0.24	0.16	0.00
	11n2	0.21	0.32	0.30	0.40	0.42	0.26	0.24	0.03
Close direct repeats	4n6g12	0.45	0.58	0.48	0.82	0.55	0.73	0.31	0.50
	6n6g24	1.35	1.16	0.71	0.34	1.00	1.21	0.81	1.39
	8n4g24	2.02	1.61	0.80	0.28	1.27	1.69	1.18	1.13
	cd8g6	0.39	0.73	0.80	1.20	0.87	0.82	0.41	0.70
	cd10g50	0.46	0.41	0.05	-0.07	0.25	0.60	0.01	0.07
Palindromes & inverted repeats	cp8g6	3.57	2.78	2.81	1.99	2.98	2.39	3.21	3.22
	cp10g50	3.56	3.26	2.98	1.64	3.15	3.03	3.45	3.20
	pals9	3.39	2.58	2.13	0.71	2.47	2.27	2.89	2.47
	pals9g12	3.83	3.46	3.05	1.79	3.33	3.22	3.55	3.64
	pals12g20	3.72	3.58	2.97	1.17	3.16	3.47	3.62	3.00
H-DNA-related patterns	cm8g6	0.02	0.20	0.21	0.33	0.24	0.24	0.15	0.04
	cm10g50	0.02	0.16	0.10	0.15	0.16	0.18	0.10	0.00
	mirs9	0.00	0.13	0.19	0.17	0.14	0.18	0.08	-0.02
	mirs9g12	0.15	0.30	0.35	0.44	0.33	0.38	0.20	0.00
	mirs12g20	0.15	0.27	0.28	0.20	0.26	0.33	0.16	0.22
	R15	-0.47	-0.52	-0.96	-1.77	-0.91	-0.55	-0.34	-1.78
	R30	0.03	0.07	0.08	0.11	0.10	0.04	0.07	0.00
	R30e3	0.08	-0.01	-0.18	-0.66	-0.23	0.00	0.14	-0.66
	R45e6	0.17	0.08	-0.03	-0.32	-0.06	0.10	0.15	-0.02
	R60e9	0.15	0.13	0.11	-0.03	0.14	0.10	0.12	0.00
G-DNA-related patterns	GG8g4	0.22	0.72	0.24	0.14	0.20	1.03	0.48	0.44
	GGG4g6	-0.08	-0.26	-0.78	-0.65	-0.67	-0.18	-0.26	-0.22
	GGGG4g6	0.00	0.10	-0.06	-0.12	0.01	0.11	0.02	0.11
Z-DNA-related patterns	GC6	-1.37	-1.50	-1.35	-0.95	-1.41	-1.38	-1.71	-1.42
	GC8	-0.75	-1.10	-0.84	-0.18	-0.47	-1.42	-1.13	-0.37
	RY12	-1.79	-1.58	-0.88	-0.16	-0.78	-1.73	-2.03	-0.99
	RY12e1	-1.81	-1.40	-0.71	-0.31	-0.85	-1.43	-1.93	-0.44
	RY18e2	-1.66	-1.45	-0.72	-0.04	-0.58	-1.60	-2.07	-0.37
	RY24e3	-0.60	-0.78	-0.25	0.05	-0.30	-0.79	-1.12	-0.44
DNA Bending	bend45w60	0.60	0.93	1.51	1.11	1.57	0.59	0.85	1.70
	bend60w100	0.32	0.79	1.40	1.17	1.42	0.50	0.74	1.48
	bend90w120	0.28	0.58	1.23	0.94	1.11	0.34	0.66	1.44

Numbers in the table refer to the average significance category for all genera within each class of organisms. Numbers at the second row indicate numbers of available genera (n) included in each class. Anaerobe includes both obligate anaerobes and anaerobes; Aerobe includes both obligate aerobes and aerobes.

Table S13. Representation of sequence patterns in different OGT and oxygen requirement classes restricted to protein coding regions

Pattern name	Pattern code	Psychrophile	Mesophile	Thermophile	Hyperthermophile	Anaerobe	Aerobe	Facultative	Microaerophile
		18	382	72	26	159	202	95	9
Simple sequence repeats	1n8	-3.76	-3.39	-3.83	-3.92	-3.73	-3.34	-3.44	-3.78
	2n5	-2.91	-2.47	-3.21	-3.09	-2.96	-2.32	-2.67	-3.00
	3n4	-1.95	-1.82	-2.71	-2.91	-2.43	-1.65	-2.07	-2.30
	4n4	0.00	0.00	-0.01	0.00	-0.02	0.01	0.00	-0.05
	5n4	0.00	0.01	0.00	0.00	0.00	0.02	0.01	0.00
	6n3	0.00	0.11	0.00	0.01	0.01	0.14	0.02	0.11
	7n3	0.00	0.07	0.07	0.00	0.07	0.08	0.04	0.00
	8n3	0.00	0.04	0.00	0.00	0.02	0.04	0.03	0.00
	9n2	-0.20	-0.02	-0.04	0.05	-0.03	0.04	-0.19	-0.11
	10n2	0.00	0.04	0.01	0.05	0.03	0.03	0.05	0.11
	11n2	0.02	0.04	0.01	0.00	0.05	0.03	0.05	0.02
Close direct repeats	4n6g12	0.07	0.29	0.19	0.53	0.22	0.40	0.20	0.07
	6n6g24	0.73	0.72	0.26	0.26	0.46	0.83	0.40	0.70
	8n4g24	1.20	0.92	0.40	0.19	0.62	1.07	0.63	0.46
	cd8g6	-0.32	0.19	0.33	0.70	0.27	0.32	-0.07	0.28
	cd10g50	-0.90	-0.36	-0.70	-0.66	-0.52	-0.20	-0.73	-0.47
Palindromes & inverted repeats	cp8g6	0.31	0.09	0.19	0.45	0.18	0.03	0.14	0.12
	cp10g50	0.28	-0.04	0.24	0.38	0.04	-0.06	0.15	0.48
	pals9	0.33	0.15	0.16	0.25	0.14	0.16	0.17	0.22
	pals9g12	0.67	0.28	0.41	0.41	0.37	0.25	0.35	0.52
	pals12g20	0.33	0.07	0.09	0.31	0.00	0.11	0.17	0.56
H-DNA-related patterns	cm8g6	0.11	0.22	0.18	0.27	0.25	0.24	0.19	0.13
	cm10g50	1.55	1.65	1.32	1.13	1.89	1.29	1.73	1.17
	mirs9	0.00	0.06	0.03	0.09	0.05	0.08	0.05	0.00
	mirs9g12	0.30	0.52	0.44	0.50	0.63	0.43	0.53	0.37
	mirs12g20	1.50	1.49	1.39	1.19	1.78	1.23	1.55	1.13
	R15	-0.40	-0.50	-1.12	-1.81	-1.09	-0.44	-0.25	-1.63
	R30	0.00	0.03	0.07	0.12	0.08	0.02	0.02	0.00
	R30e3	0.00	-0.15	-0.35	-0.82	-0.47	-0.08	0.03	-0.52
	R45e6	0.08	0.00	-0.05	-0.31	-0.14	0.04	0.08	-0.03
	R60e9	0.17	0.06	0.03	-0.07	0.03	0.06	0.08	0.00
G-DNA-related patterns	GG8g4	0.32	0.75	0.38	0.42	0.24	1.07	0.57	0.44
	GGG4g6	-0.06	-0.09	-0.39	-0.27	-0.38	0.02	-0.07	-0.11
	GGGG4g6	0.00	0.04	-0.02	-0.04	0.00	0.06	0.01	0.00
Z-DNA-related patterns	GC6	-1.05	-1.20	-1.09	-0.66	-1.10	-1.10	-1.44	-0.94
	GC8	-0.53	-0.92	-0.61	-0.25	-0.34	-1.17	-1.05	-0.13
	RY12	-1.14	-1.22	-0.65	-0.20	-0.60	-1.27	-1.71	-0.52
	RY12e1	-1.59	-1.27	-0.74	-0.43	-0.87	-1.24	-1.83	-0.05
	RY18e2	-0.93	-1.21	-0.45	-0.20	-0.56	-1.17	-1.75	-0.11
	RY24e3	-0.33	-0.60	-0.22	-0.11	-0.26	-0.55	-0.95	-0.22
DNA bending	bend45w60	0.20	0.36	1.12	1.03	0.91	0.21	0.38	1.02
	bend60w100	0.02	0.26	1.08	0.98	0.82	0.15	0.17	1.12
	bend90w120	-0.25	0.15	0.85	0.71	0.62	0.04	0.14	0.75

Same as Table S12 but showing the data for protein-coding regions only. Protein-coding regions are defined as all segments annotated as protein coding sequences (the “CDS” feature in the GenBank entry), whereas all other segments are considered non-coding. That is, untranslated regions of genes as well as RNA genes are considered “intergenic” for the purpose of this analysis.

Table S14. Representation of sequence patterns in different OGT and oxygen requirement classes restricted to the intergenic regions

Pattern name	Pattern code	Psychrophile	Mesophile	Thermophile	Hyperthermophile	Anaerobe	Aerobe	Facultative	Microaerophile
		18	382	72	26	159	202	95	9
Simple sequence repeats	1n8	-1.65	-2.14	-3.10	-3.66	-2.39	-2.48	-2.17	-2.78
	2n5	-2.05	-1.74	-2.37	-2.35	-2.08	-1.70	-1.88	-2.38
	3n4	-0.37	-0.29	-0.60	-0.61	-0.62	-0.17	-0.28	-0.35
	4n4	0.06	0.05	-0.02	0.01	0.04	0.02	0.04	0.00
	5n4	0.06	0.04	0.00	0.00	0.02	0.03	0.02	0.00
	6n3	0.36	0.18	0.07	0.00	0.09	0.18	0.14	0.44
	7n3	1.23	0.47	0.38	0.00	0.36	0.60	0.33	0.78
	8n3	0.56	0.27	0.09	0.01	0.20	0.28	0.17	0.44
	9n2	0.18	0.34	0.35	0.33	0.36	0.33	0.19	0.22
	10n2	0.15	0.29	0.32	0.19	0.31	0.28	0.20	0.00
	11n2	0.19	0.39	0.43	0.34	0.45	0.41	0.25	0.01
Close direct repeats	4n6g12	0.73	0.50	0.48	0.42	0.57	0.53	0.29	0.66
	6n6g24	1.15	0.68	0.43	0.04	0.57	0.74	0.54	0.69
	8n4g24	1.32	0.99	0.62	0.04	0.84	1.05	0.72	0.78
	cd8g6	1.54	1.33	1.26	1.25	1.56	1.31	0.93	1.16
	cd10g50	2.13	1.84	1.64	1.05	1.88	1.88	1.52	1.46
Palindromes & inverted repeats	cp8g6	3.89	3.78	3.49	2.36	3.56	3.68	3.82	3.89
	cp10g50	3.89	3.83	3.37	1.94	3.49	3.76	3.79	3.67
	pals9	2.29	1.40	0.94	0.20	1.18	1.26	1.79	0.88
	pals9g12	3.72	3.49	2.72	1.06	2.96	3.38	3.65	2.79
	pals12g20	2.84	2.04	1.28	0.31	1.62	1.95	2.40	1.02
H-DNA-related patterns	cm8g6	0.10	0.20	0.17	0.26	0.26	0.17	0.12	0.23
	cm10g50	-0.11	0.15	0.14	0.03	0.16	0.14	0.07	0.15
	mirs9	0.00	0.01	0.07	0.00	0.04	0.01	0.01	0.00
	mirs9g12	0.06	0.10	0.09	0.04	0.12	0.09	0.03	0.00
	mirs12g20	0.00	0.01	0.04	0.00	0.04	0.01	0.00	0.00
	R15	-0.15	0.08	0.28	0.07	0.21	-0.05	0.16	-0.18
	R30	0.00	0.03	0.02	0.04	0.05	0.02	0.05	0.00
	R30e3	0.24	0.17	0.17	0.25	0.19	0.12	0.19	0.55
	R45e6	0.21	0.14	0.18	0.09	0.12	0.12	0.19	0.33
	R60e9	0.06	0.09	0.19	0.10	0.10	0.09	0.11	0.33
G-DNA-related patterns	GG8g4	0.08	0.12	0.05	0.12	0.03	0.15	0.09	0.33
	GGG4g6	-0.06	-0.17	-0.54	-0.55	-0.28	-0.30	-0.12	-0.11
	GGGG4g6	0.06	0.07	0.00	-0.04	0.03	0.08	0.05	0.00
Z-DNA-related patterns	GC6	-1.03	-1.17	-0.79	-0.30	-0.71	-1.36	-1.03	-0.73
	GC8	-0.29	-0.48	-0.37	0.01	-0.14	-0.74	-0.36	-0.04
	RY12	-1.09	-0.64	-0.18	-0.19	-0.16	-0.83	-0.76	-0.48
	RY12e1	-1.86	-1.20	-0.22	-0.06	-0.22	-1.48	-1.57	-0.59
	RY18e2	-0.95	-0.57	-0.21	0.06	-0.05	-0.78	-0.81	-0.47
	RY24e3	-0.08	-0.11	0.02	0.18	0.08	-0.16	-0.21	-0.35
DNA bending	bend45w60	1.02	0.92	0.77	0.28	1.03	0.66	0.94	1.72
	bend60w100	0.85	0.74	0.56	0.36	0.86	0.53	0.76	1.29
	bend90w120	0.41	0.52	0.40	0.28	0.68	0.29	0.54	0.89

Same as Table S12 but showing the data for 'intergenic' regions only, that is, all regions not annotated as 'CDS' in the GenBank files.

Table S15. Representation of sequence patterns in different oxygen requirement classes restricted to mesophilic organism

Pattern name	Pattern code	All Mesophile	Anaerobe	Aerobe	Facultative	Microaerophile
		382	98	168	81	5
Simple sequence repeats	1n8	-3.54	-3.59	-3.58	-3.60	-3.40
	2n5	-2.89	-3.13	-2.70	-2.98	-3.20
	3n4	-1.92	-2.25	-1.63	-2.10	-2.40
	4n4	0.01	0.00	0.01	0.00	-0.18
	5n4	0.07	0.03	0.11	0.02	0.20
	6n3	0.19	0.08	0.26	0.07	0.37
	7n3	0.48	0.41	0.62	0.30	0.80
	8n3	0.30	0.33	0.30	0.20	0.20
	9n2	0.09	0.07	0.17	-0.12	0.00
	10n2	0.25	0.27	0.25	0.17	0.00
	11n2	0.32	0.40	0.28	0.23	0.05
Close direct repeats	4n6g12	0.58	0.53	0.74	0.30	0.71
	6n6g24	1.16	1.20	1.27	0.82	1.71
	8n4g24	1.61	1.61	1.81	1.21	1.43
	cd8g6	0.73	0.89	0.78	0.41	0.66
	cd10g50	0.41	0.49	0.60	0.00	0.53
Palindromes & inverted repeats	cp8g6	2.78	3.03	2.34	3.34	3.20
	cp10g50	3.26	3.42	3.01	3.65	3.77
	pals9	2.58	2.87	2.29	3.00	2.84
	pals9g12	3.46	3.63	3.23	3.73	3.94
	pals12g20	3.58	3.54	3.54	3.84	3.80
H-DNA-related patterns	cm8g6	0.20	0.24	0.23	0.14	0.07
	cm10g50	0.16	0.19	0.19	0.10	0.00
	mirs9	0.13	0.11	0.20	0.04	-0.03
	mirs9g12	0.30	0.30	0.38	0.16	0.00
	mirs12g20	0.27	0.27	0.35	0.12	0.20
	R15	-0.52	-0.51	-0.54	-0.40	-2.00
	R30	0.07	0.12	0.04	0.03	0.00
	R30e3	-0.01	-0.02	0.02	0.06	-0.99
	R45e6	0.08	0.04	0.11	0.11	0.16
	R60e9	0.13	0.20	0.12	0.08	0.00
G-DNA-related patterns	GG8g4	0.72	0.26	1.11	0.52	0.20
	GGG4g6	-0.26	-0.55	-0.13	-0.27	0.01
	GGGG4g6	0.10	0.07	0.16	0.02	0.20
Z-DNA-related patterns	GC6	-1.50	-1.63	-1.34	-1.73	-2.35
	GC8	-1.10	-0.60	-1.37	-1.25	-0.47
	RY12	-1.58	-0.92	-1.78	-2.21	-1.38
	RY12e1	-1.40	-0.97	-1.47	-2.15	-1.20
	RY18e2	-1.45	-0.71	-1.64	-2.32	-0.67
	RY24e3	-0.78	-0.39	-0.84	-1.31	-0.80
DNA bending	bend45w60	0.93	1.52	0.62	0.81	2.06
	bend60w100	0.79	1.30	0.52	0.70	1.66
	bend90w120	0.58	0.94	0.35	0.63	1.40

Only mesophiles were included in this table in order to assess the independence of trends with respect to OGT and oxygen requirement. See **Error! Reference source not found.**.

Supplementary Table S16. Representation of sequence patterns in different oxygen requirement classes restricted to thermophilic organism

Pattern name	Pattern code	Thermophile	Anaerobe	Aerobe	Facultative	Microaerophile
		112	61	26	13	4
Simple sequence repeats	1n8	-3.89	-3.94	-3.77	-3.92	-4.00
	2n5	-3.40	-3.59	-3.01	-3.24	-3.00
	3n4	-2.83	-3.15	-2.27	-2.48	-2.25
	4n4	-0.06	-0.10	0.00	0.00	0.00
	5n4	0.00	0.00	0.00	0.00	0.00
	6n3	0.06	0.00	0.08	0.04	0.25
	7n3	0.45	0.34	0.12	0.35	1.00
	8n3	0.12	0.07	0.04	0.12	0.50
	9n2	0.22	0.12	0.38	0.00	-0.25
	10n2	0.25	0.31	0.23	0.12	0.00
	11n2	0.30	0.50	0.12	0.24	0.00
Close direct repeats	4n6g12	0.48	0.57	0.69	0.42	0.25
	6n6g24	0.71	0.63	0.50	0.42	1.00
	8n4g24	0.80	0.66	0.66	0.70	0.75
	cd8g6	0.80	0.84	1.14	0.50	0.75
	cd10g50	0.05	-0.17	0.58	-0.14	-0.50
Palindromes & inverted repeats	cp8g6	2.81	2.79	2.06	2.12	3.25
	cp10g50	2.98	2.67	3.07	2.00	2.50
	pals9	2.13	1.73	1.80	1.88	2.00
	pals9g12	3.05	2.80	2.89	2.08	3.25
	pals12g20	2.97	2.52	2.98	1.92	2.00
H-DNA-related patterns	cm8g6	0.21	0.26	0.27	0.08	0.00
	cm10g50	0.10	0.12	0.13	0.04	0.00
	mirs9	0.19	0.19	0.16	0.23	0.00
	mirs9g12	0.35	0.37	0.38	0.29	0.00
	mirs12g20	0.28	0.24	0.25	0.27	0.25
	R15	-0.96	-1.58	-0.60	0.18	-1.50
	R30	0.08	0.09	0.01	0.32	0.00
	R30e3	-0.18	-0.65	-0.07	1.02	-0.25
	R45e6	-0.03	-0.24	0.06	0.45	-0.25
	R60e9	0.11	0.03	0.06	0.30	0.00
G-DNA-related patterns	GG8g4	0.24	0.07	0.62	0.27	0.75
	GGG4g6	-0.78	-0.90	-0.50	-0.59	-0.50
	GGGG4g6	-0.06	-0.07	-0.12	0.04	0.00
Z-DNA-related patterns	GC6	-1.35	-1.11	-1.66	-1.66	-0.25
	GC8	-0.84	-0.29	-1.94	-0.44	-0.25
	RY12	-0.88	-0.50	-1.54	-0.26	-0.50
	RY12e1	-0.71	-0.60	-1.09	-0.13	0.50
	RY18e2	-0.72	-0.32	-1.45	-0.14	0.00
	RY24e3	-0.25	-0.13	-0.51	0.10	0.00
DNA bending	bend45w60	1.51	1.70	0.49	1.59	1.25
	bend60w100	1.40	1.63	0.44	1.56	1.25
	bend90w120	1.23	1.42	0.34	1.31	1.50

Same as Table S15 but including only thermophiles.

Table S17. Representation of sequence patterns in different oxygen requirement classes restricted to bacteria

Pattern name	Pattern code	Anaerobe	Aerobe	Facultative	Microaerophile
		122	189	90	9
Simple sequence repeats	1n8	-3.73	-3.60	-3.64	-3.67
	2n5	-3.27	-2.72	-3.01	-3.11
	3n4	-2.48	-1.70	-2.10	-2.33
	4n4	-0.03	0.01	0.00	-0.10
	5n4	0.02	0.10	0.01	0.11
	6n3	0.05	0.24	0.08	0.31
	7n3	0.52	0.61	0.37	0.89
	8n3	0.26	0.27	0.22	0.33
	9n2	0.03	0.18	-0.11	-0.11
	10n2	0.19	0.23	0.16	0.00
	11n2	0.36	0.25	0.25	0.03
Close direct repeats	4n6g12	0.45	0.74	0.33	0.50
	6n6g24	1.07	1.19	0.84	1.39
	8n4g24	1.38	1.70	1.22	1.13
	cd8g6	0.74	0.80	0.42	0.70
	cd10g50	0.31	0.59	0.05	0.07
Palindromes & inverted repeats	cp8g6	3.24	2.41	3.31	3.22
	cp10g50	3.65	3.16	3.58	3.20
	pals9	3.00	2.41	3.03	2.47
	pals9g12	3.76	3.32	3.65	3.64
	pals12g20	3.79	3.65	3.77	3.00
H-DNA-related patterns	cm8g6	0.15	0.23	0.16	0.04
	cm10g50	0.12	0.18	0.11	0.00
	mirs9	0.06	0.19	0.08	-0.02
	mirs9g12	0.24	0.38	0.21	0.00
	mirs12g20	0.21	0.33	0.17	0.22
	R15	-0.78	-0.49	-0.36	-1.78
	R30	0.10	0.03	0.04	0.00
	R30e3	-0.13	0.02	0.12	-0.66
	R45e6	-0.03	0.10	0.14	-0.02
	R60e9	0.15	0.10	0.10	0.00
G-DNA-related patterns	GG8g4	0.23	1.10	0.52	0.44
	GGG4g6	-0.73	-0.13	-0.23	-0.22
	GGGG4g6	-0.01	0.13	0.02	0.11
Z-DNA-related patterns	GC6	-1.45	-1.33	-1.70	-1.42
	GC8	-0.52	-1.40	-1.14	-0.37
	RY12	-0.93	-1.73	-2.11	-0.99
	RY12e1	-1.03	-1.42	-2.01	-0.44
	RY18e2	-0.73	-1.63	-2.14	-0.37
	RY24e3	-0.39	-0.83	-1.18	-0.44
DNA bending	bend45w60	1.51	0.59	0.79	1.70
	bend60w100	1.33	0.49	0.69	1.48
	bend90w120	1.01	0.32	0.63	1.44

Archaeal genomes were excluded from the data used to generate this table. See **Error! Reference source not found.** for legend.

Table S18. Number of ATG and GTG start codons embedded in RY-patterns in selected genomes

Species	N_{ATG_RY}	N_{ATG}	Proportion	$N_{\overline{ATG\_RY}}$	$N_{\overline{ATG}}$	Proportion	N_{GTG_RY}	N_{GTG}	Proportion	$N_{\overline{GTG\_RY}}$	$N_{\overline{GTG}}$	Proportion
Chlamydophila pecorum	57	813	7.01%	2,301	35,043	6.57%	0	83	0.00%	1,135	18,798	6.04%
Escherichia coli	154	3,702	4.16%	7,189	149,521	4.81%	11	307	3.58%	5,836	127,565	4.57%
Helicobacter pylori	32	1,282	2.50%	2,955	55,264	5.35%	0	105	0.00%	1,592	34,012	4.68%
Helicobacter felis	96	1,361	7.05%	4,574	55,901	8.18%	11	149	7.38%	4,110	46,662	8.81%
Helicobacter acinonychis	39	1,216	3.21%	3,144	52,483	5.99%	6	143	4.20%	1,811	34,295	5.28%
Helicobacter bizzozeronii	76	1,365	5.57%	4,336	61,445	7.06%	8	227	3.52%	3,581	52,866	6.77%
Thermofilum pendens	89	1,073	8.29%	2,344	28,562	8.21%	37	562	6.58%	3,208	44,316	7.24%
Treponema brennaborense	194	2,285	8.49%	5,337	69,231	7.71%	8	164	4.88%	6,169	56,986	10.83%
Treponema pallidum	78	604	12.91%	4,740	31,469	15.06%	40	322	12.42%	7,590	42,860	17.71%

N_{ATG_RY} is the number of start codons ATG embedded in RY patterns (RY12e1, RY18e2 or RY24e3); $N_{\overline{ATG\_RY}}$ is the number of ATG triplets that are not start codons non-start codon ATG embedded in RY patterns; N_{ATG} is the total number of start codon ATG in the genome; $N_{\overline{ATG}}$ is the number of non-start codon ATG triplets presented in the RY patterns. Same as N_{GTG_RY}, $N_{\overline{GTG\_RY}}$, N_{GTG} and $N_{\overline{GTG}}$. ‘Proportion’ is the percentage of all ATG/GTG in a given category that are embedded in RY patterns.

Table S19. Mann–Whitney U-test for ratio of intrinsic DNA bends in protein-coding and non-coding regions among different OGT groups

bend60w100 \ bend45w60	Hyperthermophile	Thermophile	Mesophile	Psychrophile
**Hyperthermophile**		0.8943	0.0001	0.0203
**Thermophile**	0.8857		$< 10^{-9}$	0.0008
**Mesophile**	0.0001	$< 10^{-9}$		0.6303
**Psychrophile**	0.0036	0.0001	0.3907	

The ratio of the protein coding and non-coding bend was assessed for each genome and subsequently compared among the genomes belonging to the four OGT classes. The p-values assesed by the Mann-Whitney U test are shown in the table. Low p-values indicate significant differences in protein-coding to noncoding bend ratios between the two classes sompared. Results for the bend45w60 pattern are shown in the upper right triangle whereas those for the bend60w100 patterns are shown in the lower left side triangle.
